# Impaired T cell IRE1**α**/XBP1 signaling directs inflammation in experimental heart failure with preserved ejection fraction

**DOI:** 10.1172/JCI171874

**Published:** 2023-12-15

**Authors:** Sasha Smolgovsky, Abraham L. Bayer, Kuljeet Kaur, Erin Sanders, Mark Aronovitz, Mallory E. Filipp, Edward B. Thorp, Gabriele G. Schiattarella, Joseph A. Hill, Robert M. Blanton, Juan R. Cubillos-Ruiz, Pilar Alcaide

**Affiliations:** 1 Department of Immunology, Tufts University, Boston, Massachusetts, USA.; 2 Feinberg School of Medicine, Northwestern University, Chicago, Illinois, USA.; 3 Max Rubner Center for Cardiovascular Metabolic Renal Research (MRC), Deutsches Herzzentrum der Charité, Charité – Universitätsmedizin Berlin, Berlin, Germany.; 4 DZHK (German Centre for Cardiovascular Research), Partner Site Berlin, Berlin, Germany.; 5 Translational Approaches in Heart Failure and Cardiometabolic Disease, Max Delbrück Center for Molecular Medicine in the Helmholtz Association, Berlin, Germany.; 6 Department of Internal Medicine (Cardiology) and; 7 Department of Molecular Biology, UT Southwestern Medical Center, Dallas, Texas, USA.; 8 Molecular Cardiology Research Institute, Tufts Medical Center, Boston, Massachusetts, USA.; 9 Department of Obstetrics and Gynecology and; 10 Sandra and Edward Meyer Cancer Center, Weill Cornell Medicine, New York, New York, USA.; 11 Weill Cornell Graduate School of Medical Sciences, New York, New York, USA.

**Keywords:** Cardiology, Inflammation, Adaptive immunity, Cardiovascular disease, Heart failure

## Abstract

Heart failure with preserved ejection fraction (HFpEF) is a widespread syndrome with limited therapeutic options and poorly understood immune pathophysiology. Using a 2-hit preclinical model of cardiometabolic HFpEF that induces obesity and hypertension, we found that cardiac T cell infiltration and lymphoid expansion occurred concomitantly with cardiac pathology and that diastolic dysfunction, cardiomyocyte hypertrophy, and cardiac phospholamban phosphorylation were T cell dependent. Heart-infiltrating T cells were not restricted to cardiac antigens and were uniquely characterized by impaired activation of the inositol-requiring enzyme 1α/X-box–binding protein 1 (IRE1α/XBP1) arm of the unfolded protein response. Notably, selective ablation of XBP1 in T cells enhanced their persistence in the heart and lymphoid organs of mice with preclinical HFpEF. Furthermore, T cell IRE1α/XBP1 activation was restored after withdrawal of the 2 comorbidities inducing HFpEF, resulting in partial improvement of cardiac pathology. Our results demonstrated that diastolic dysfunction and cardiomyocyte hypertrophy in preclinical HFpEF were T cell dependent and that reversible dysregulation of the T cell IRE1α/XBP1 axis was a T cell signature of HFpEF.

## Graphical abstract

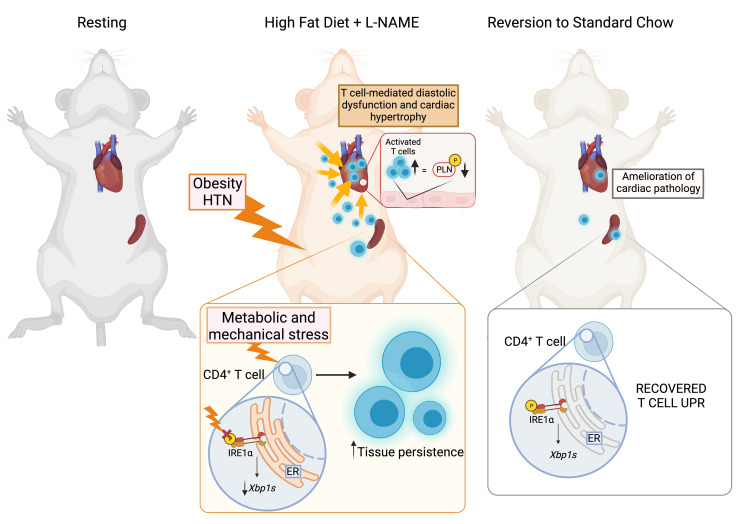

## Introduction

Widely regarded as the greatest unmet need in cardiovascular medicine, heart failure with preserved ejection fraction (HFpEF) is a complex and heterogeneous syndrome accounting for roughly 50% of HF diagnoses, with increasing prevalence and limited therapeutic options (1). Whereas multiple therapies have been developed for HF with reduced ejection fraction (HFrEF), few of these show significant benefit in HFpEF patients, likely owing to cellular and molecular mechanisms that differentiate these 2 conditions (2). Patients with cardiometabolic HFpEF, a subset of HFpEF conditions with rising global prevalence, present with several comorbidities, including obesity and hypertension, which each trigger independent inflammatory mechanisms (3). Consistent with this, clinical data demonstrate an increase in circulating proinflammatory cytokines and elevated circulating T cells in HFpEF patients (4, 5), suggesting contributions of active adaptive immune responses. Additionally, RNA sequencing from HFpEF patient endomyocardial biopsies reveals immune response pathways to be enriched in comparison with healthy controls (6). However, whereas T cell inflammation and the mechanisms contributing to adverse cardiac remodeling in various HFrEF etiologies have been established in the past decade (7–9), mechanistic studies interrogating the type of T cell immune response and T cell contributions to cardiac pathology and diastolic dysfunction in HFpEF are lacking.

In HFrEF, the pathophysiology usually arises from a cardiac insult, causing a primary defect in cardiac structure and function. Cardiac immune cells, such as cardiac antigen–specific T cells, contribute to this “inside-out” process, being recruited to the heart in response to these insults and driving key aspects of ventricular dysfunction and remodeling (10, 11). Conversely, cardiac pathology in HFpEF arises initially from systemic, extracardiac tissue abnormalities in an “outside-in” manner. This is supported by clinical data revealing that HFpEF patients have increased circulating cytokines that are not sourced from the myocardium, suggesting the presence of extracardiac sources of inflammation (12). From the immune standpoint, in response to long-standing metabolic perturbations induced by systemic comorbidities, T cells are expanded in the periphery in response to stressors originating outside of the heart, such as metabolic stress induced by 2 of the most frequent risk factors of HFpEF, obesity and hypertension. However, the contribution of peripheral T cell expansion to direct cardiac dysfunction has not been investigated.

Disruption of endoplasmic reticulum (ER) homeostasis triggers the unfolded protein response (UPR) (13). Inositol-requiring enzyme-1α (IRE1α)–X-box binding protein 1 (XBP1) is a major arm of the UPR and the most evolutionarily conserved cellular axis implicated in restoring ER proteostasis. This arm was recently shown to be downregulated in the myocardium of HFpEF patients and a cardiac hallmark distinguishing HFpEF from HFrEF. In mice subjected to experimental cardiometabolic HFpEF induced by 2 hits, high-fat diet (HFD) and l-NAME, to mimic combined obesity and hypertension, overexpression of XBP1 in cardiomyocytes partially ameliorated diastolic function (14). Dysregulation of the UPR has further been described in T cells in the context of cancer, and T cell–intrinsic XBP1 deficiency in particular was shown to enhance adaptive antitumor immunity (15, 16).

Here, using the 2-hit model of preclinical HFpEF, we tested the hypothesis that T cells contribute to diastolic dysfunction in cardiometabolic HFpEF and identified a possible unique T cell signature characterized by a defective IRE1α-XBP1 activation.

## Results

### HFD/l-NAME elicits cardiac T cell inflammation concordant with cardiac pathology.

**Figure 1:**
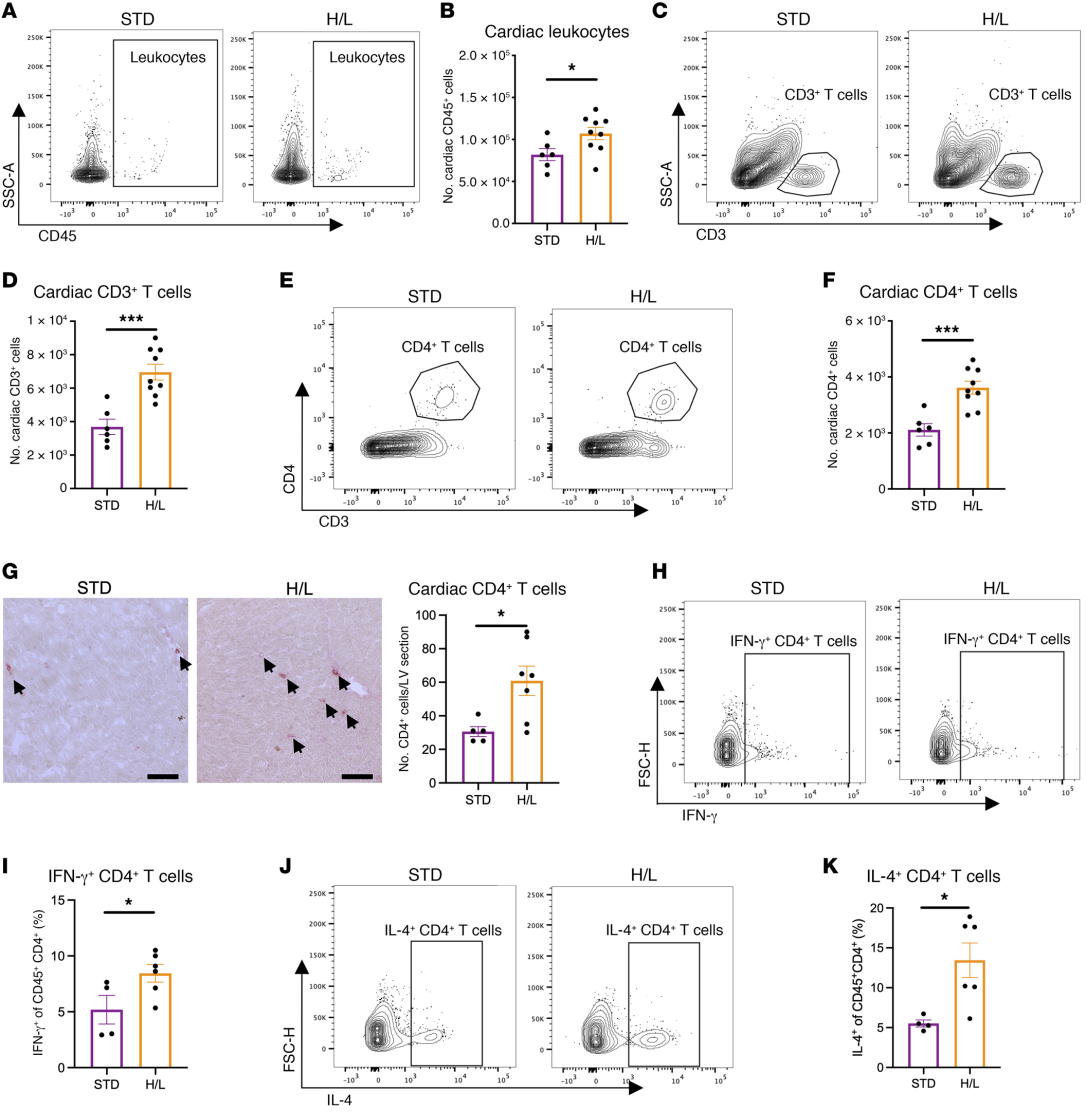
H/L elicits increased leukocyte and T cell myocardial infiltration. (**A**–**F**) Cardiac CD45^+^ (**A** and **B**), CD45^+^CD3^+^ (**C** and **D**), and CD45^+^CD3^+^CD4^+^ (**E** and **F**) cells were directly analyzed by flow cytometry from LV of WT mice fed H/L or STD for 5 weeks. (**G**) CD4^+^ T cells were immunohistochemically stained and quantified in LV cryosections of mice from each group. Scale bars: 50 μm. (**H**–**K**) CD45^+^CD4^+^IFN-γ^+^ (**H** and **I**) and CD45^+^CD4^+^IL-4^+^ (**J** and **K**) cells were directly analyzed by flow cytometry from LV of WT mice fed H/L or STD for 5 weeks. *n* = 4–9. Error bars represent mean ± SEM. Unpaired, 2-tailed *t* test. *P ≤ 0.05, ***P ≤ 0.001. This figure was created using Biorender.com.

We first examined the cardiac and systemic immune phenotype in cardiometabolic HFpEF after 5 weeks of HFD/l-NAME (H/L). Flow cytometry analysis revealed a significant increase in cardiac CD45^+^ leukocytes in H/L-fed mice compared with mice fed standard chow (STD) (Figure 1, A and B, and Supplemental Figure 1A; supplemental material available online with this article; https://doi.org/10.1172/JCI171874DS1). Several leukocyte populations were specifically increased in the left ventricle (LV) in response to H/L compared with STD, including CD3^+^ T cells (Figure 1, C and D) and CD4^+^ T cells (Figure 1, E and F). Histological analysis identified CD4^+^ T cells infiltrating the interstitial myocardium (Figure 1G). Other lymphocytes such as CD8^+^ T cells were also increased, although to a lesser extent (Supplemental Figure 1B), as were CD11b^+^ myeloid cells, including monocytes and neutrophils (Supplemental Figure 1, C–F). Additionally, H/L increased myocardial gene expression of NLR family pyrin domain containing 3 (NLRP3), an intracellular sensor and inducer of pyroptotic cell death (17), supporting an enhanced inflammatory state of the heart that may be responsible for recruiting innate immune cells to the myocardium (Supplemental Figure 1G). Additionally, H/L increased myocardial B cell infiltration (Supplemental Figure 1H). In further characterization of cardiac T helper cells, we used intracellular staining of cardiac CD4^+^ T cells and found enrichment of CD4^+^ T cells expressing interferon-γ (IFN-γ; Figure 1, H and I) and interleukin-4 (IL-4; Figure 1, J and K) in response to H/L, in comparison with control-fed mice. We observed no significant increases in cardiac CD4^+^ T cells expressing IL-17A following H/L compared with STD controls (Supplemental Figure 1, I and J).

**Figure 2:**
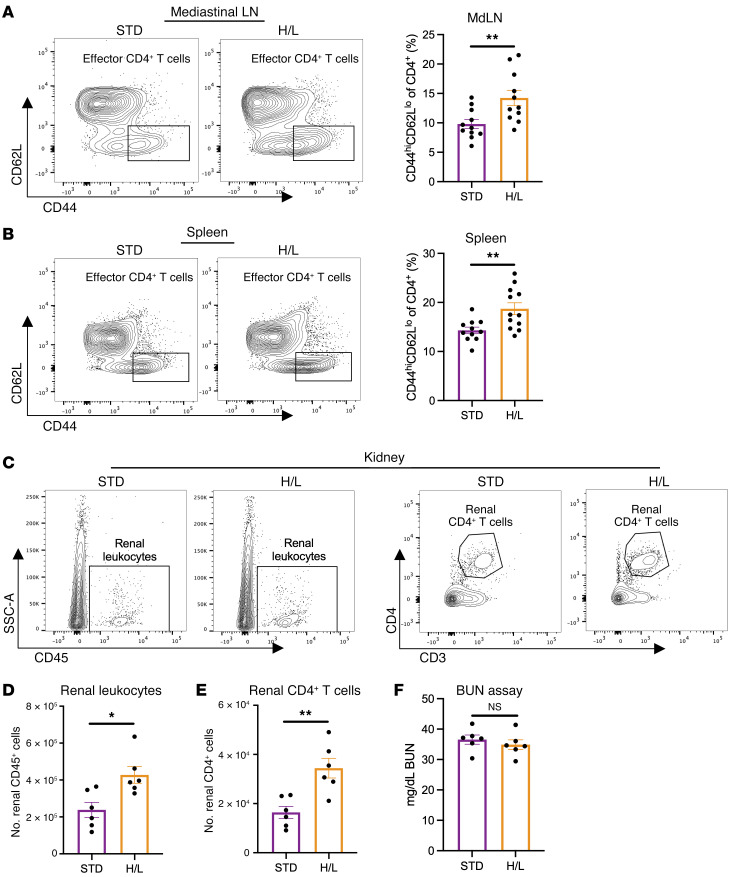
H/L induces lymphoid T cell expansion and increased renal T cell infiltration. CD4^+^CD44^hi^CD62L^lo^ effector T cells in mediastinal lymph nodes (MdLN) (**A**) or spleen (**B**) and renal CD45^+^ cells and CD4^+^ cells (**C**–**E**) were analyzed by flow cytometry, and renal function was assessed with blood urea nitrogen (BUN) assay (**F**), in WT mice fed for 5 weeks with H/L or STD. *n* = 6–12. Error bars represent mean ± SEM. Unpaired, 2-tailed *t* test. *P ≤ 0.05, **P ≤ 0.01. This figure was created using Biorender.com.

The observed cardiac T cell inflammation occurred concomitant with the presence of effector CD4^+^ T cells in the local and distal lymphoid organs, such as mediastinal lymph nodes (MdLNs) and the spleen, which showed CD4^+^CD44^hi^CD62^lo^ cell expansion in response to H/L but not in STD controls (Figure 2, A and B, and Supplemental Figure 1K). This effector T cell expansion occurred in H/L-fed mice but not in the MdLNs and the spleen of HFD– or l-NAME–single-treated controls (Supplemental Figure 1, L and M). In contrast to treatment of mice with H/L, HFD in isolation induced neither hypertrophy nor significant CD45^+^ and CD4^+^ LV cell infiltration, compared with STD, whereas exclusive exposure to l-NAME did not cause LV hypertrophy compared with STD, despite triggering increases in CD45^+^ and CD4^+^ cardiac-infiltrating cells (Supplemental Figure 1, N–P). Females, which are protected in this preclinical HFpEF model (18) (Supplemental Figure 1, Q and R, and Supplemental Table 1), also showed enhanced cardiac CD4^+^ T cell infiltration and splenic effector CD4^+^ T cell expansion in response to H/L- compared with STD-fed mice (Supplemental Figure 1, S and T). These data suggest that in females there are mechanisms in place to protect the myocardium from developing diastolic dysfunction and cardiomyocyte hypertrophy, even in the presence of T cell infiltration.

As HFpEF is a systemic, multi-organ disorder (3), and chronic kidney disease and HFpEF share many comorbidities and often present together (19), we hypothesized that heightened T cell infiltration may not be restricted to the heart. Indeed, we found increased renal leukocyte and CD4^+^ T cell abundance in the kidneys of H/L-treated mice compared with kidneys from STD controls (Figure 2, C–E, and Supplemental Figure 1U). However, there were no differences in plasma levels of blood urea nitrogen (BUN), indicating a lack of renal dysfunction after 5 weeks of H/L (Figure 2F).

Taken together, these data indicate that H/L induces CD4^+^ T cell infiltration into the heart and kidneys, as well as effector CD4^+^ T cell expansion in the spleen and lymph nodes. While cardiac T cell infiltration aligns with diastolic dysfunction and cardiomyocyte hypertrophy, markers of kidney dysfunction, such as plasma BUN, are not evident despite the presence of kidney-infiltrated T cells at this time point. These data support the presence of “outside-in” inflammatory signals in HFpEF and that T cell contributions to cardiac pathology occur earlier than possible contributions to renal dysfunction in preclinical HFpEF.

### T cell–deficient (Tcra^–/–^) mice do not develop diastolic dysfunction and cardiomyocyte hypertrophy in response to H/L.

**Figure 3:**
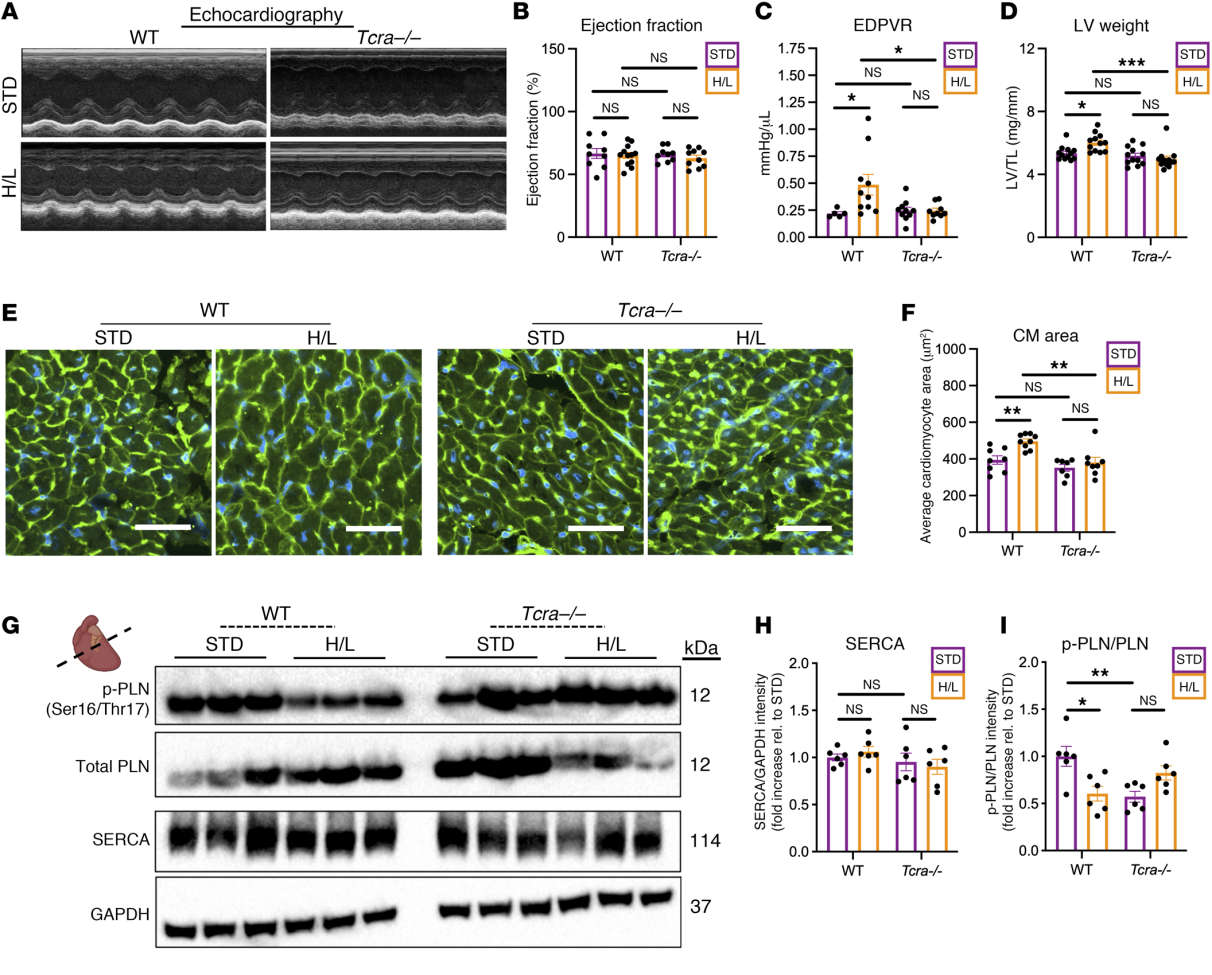
Diastolic dysfunction and cardiac hypertrophy are not induced by H/L in T cell–deficient mice. (**A**–**C**) The ejection fraction (**A** and **B**) and end-diastolic pressure–volume relationship (EDPVR; **C**) were measured in WT or *Tcra^–/–^* mice fed H/L or STD for 5 weeks using echocardiography and invasive hemodynamic analysis, respectively. (**D**) The LV weight of WT or *Tcra^–/–^* mice was measured and normalized to tibia length (LV/TL). (**E** and **F**) LV cryosections from these mice were stained with wheat germ agglutinin and analyzed for cardiomyocyte area using ImageJ. Scale bars: 50 μm. (**G**–**I**) The expression of SERCA, phospho-PLN, and total PLN was measured in LV samples using Western blotting. *n* = 5–12. Error bars represent the mean ± SEM. Two-way ANOVA with Šidák’s multiple-comparison test. *P ≤ 0.05, **P ≤ 0.01, ***P ≤ 0.001. This figure was created using Biorender.com.

We next investigated the requirement of T cells for diastolic dysfunction using T cell receptor-α–knockout (*Tcra^–/–^*) mice, which lack α/β T cells. Both groups had preserved ejection fraction (Figure 3, A and B, and Supplemental Table 2) and experienced increases in systolic blood pressure compared with STD-fed controls (Supplemental Figure 2A). Whereas 5 weeks of H/L induced, as expected, impaired relaxation in WT mice as measured by the slope of the end-diastolic pressure–volume relationship (EDPVR), *Tcra^–/–^* mice did not show such an increase, demonstrating preserved cardiac relaxation compared with WT (Figure 3C). Moreover, the cardiometabolic stress induced by H/L resulted in LV and cardiomyocyte hypertrophy in WT mice exclusively, and not in *Tcra^–/–^* mice, as shown by gross organ weight and wheat germ agglutinin staining of cardiac sections, respectively (Figure 3, D–F).

To uncover the mechanisms by which T cells contribute to diastolic dysfunction in response to H/L, we next focused on investigating the protein expression of key mediators of cardiomyocyte relaxation: sarco(endo)plasmic reticulum Ca^++^-ATPase (SERCA), responsible for cytoplasmic calcium import into the sarcoplasmic reticulum to perpetuate relaxation; and phospholamban (PLN), an inhibitor of SERCA when in an unphosphorylated state (20) (Figure 3G). We found that the ratio of phosphorylated to total PLN was decreased in the LV of WT mice fed H/L compared with STD controls, whereas LV protein expression of SERCA remained unaltered. Strikingly, the decrease in the phosphorylated to total PLN was not observed in *Tcra^–/–^* mice fed H/L compared with STD controls, whereas SERCA was also unaltered in *Tcra^–/–^* mice fed H/L or STD (Figure 3, H and I). These data suggest a greater abundance of unphosphorylated PLN that can inhibit SERCA and cardiomyocyte relaxation in mice with preclinical HFpEF that is T cell dependent, revealing that T cells modulate PLN phosphorylation in response to H/L. Moreover, we observed a trending increase in total PLN expression in WT mice, but not *Tcra^–/–^* mice, fed H/L, suggesting additional T cell–mediated regulation of PLN expression (Supplemental Figure 2B). Altogether, these findings indicate that T cell–cardiomyocyte crosstalk contributes to maladaptive cardiac remodeling in response to H/L.

### The effector T cells induced after 5 weeks of HFD and l-NAME are not cardiac antigen restricted.

**Figure 4:**
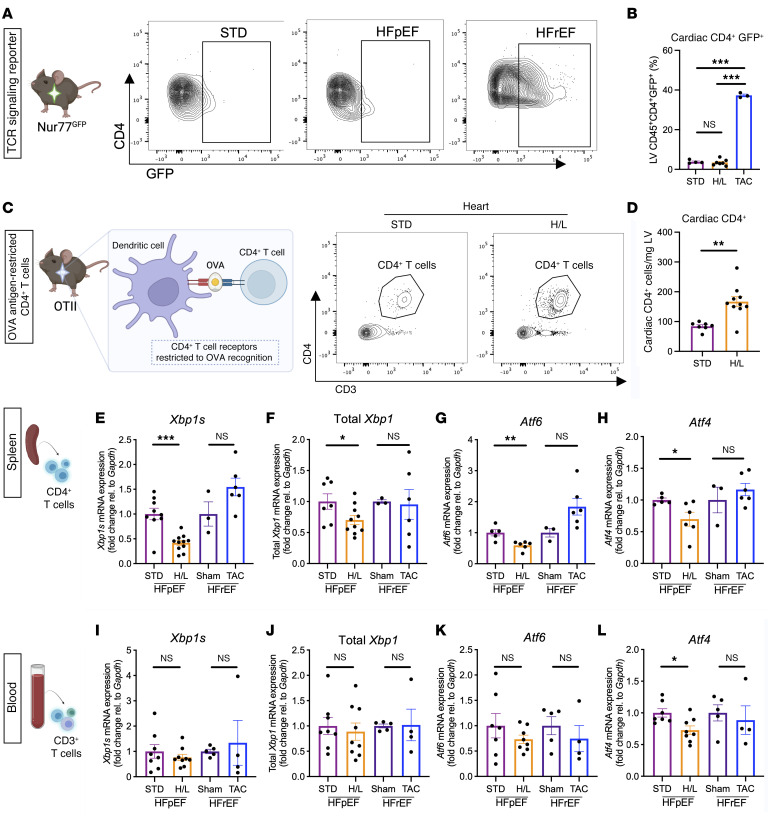
Experimental HFpEF imprints a T cell immune signature distinct to experimental HFpEF. (**A** and **B**) CD45^+^CD4^+^GFP^+^ cardiac cells were analyzed in STD, H/L, and TAC surgery Nur77^GFP^ mice 5 weeks after respective treatment. (**C** and **D**) Cardiac CD45^+^CD3^+^CD4^+^ cells were measured directly by flow cytometry in OTII mice fed H/L or STD for 5 weeks. (**E**–**L**) Splenic CD4^+^ (**E**–**H**) or blood CD3^+^ (**I**–**L**) T cells were isolated from WT mice fed H/L or STD (HFpEF), or from WT mice given TAC or sham surgery (HFrEF), and analyzed for the gene expression of *Xbp1s*, total *Xbp1*, *Atf6*, and *Atf4* by qPCR. *n* = 3–11. **I**–**L**: Each replicate is *n* = 2 mice pooled. Error bars represent the mean ± SEM. **B**: One-way ANOVA with Tukey’s multiple-comparison test; **D**: unpaired, 2-tailed *t* test; **E**–**L**: HF groups (H/L or TAC) were compared against their respective controls (STD and sham, respectively) in the unpaired, 2-tailed *t* test. *P ≤ 0.05, **P ≤ 0.01, ***P ≤ 0.001. This figure was created using Biorender.com.

We next investigated whether H/L induced a T cell response to cardiac antigen, similar to what we and others have described in experimental HFrEF (7, 11). We used Nur77^GFP^ reporter mice, in which T cells transiently express GFP upon TCR antigen engagement, yet their overall T cell repertoire and inflammatory potential remains unaltered (21). This allowed us to track active TCR antigen engagement within the heart after 5 weeks of H/L or STD control diet, using Nur77^GFP^ mice subjected to transverse aortic constriction (TAC) to model HFrEF, in which TCR engagement occurs in the heart, as positive control (7). Nur77^GFP^ mice developed increased LV weight in response to H/L, but this was independent of active CD4^+^ TCR engagement at this time point, reflected by a similar frequency of CD4^+^GFP^+^ cardiac T cells in H/L mice and STD controls, and a significantly lower frequency than in mice subjected to TAC (Figure 4, A and B, and Supplemental Figure 3, A and B). These data indicate that infiltrating CD4^+^ cells do not experience TCR engagement of antigens in the heart at the time of cardiac hypertrophy and diastolic dysfunction in experimental HFpEF, as opposed to HFrEF.

To further investigate the antigen specificity of cardiac T cell activation in HFpEF, we studied OTII mice, in which all CD4^+^ T cells express a transgenic TCR restricted to exogenous chicken ovalbumin (OVA). Thus, T cells in these mice do not become antigen activated unless they recognize OVA. OTII mice fed H/L manifested preserved ejection fraction (Supplemental Table 3), and a striking increase of cardiac CD4^+^ T cell infiltration compared with STD-fed OTII mice (Figure 4, C and D, and Supplemental Figure 3C). The cardiotropism of OVA-restricted T cells in response to H/L correlated with increased systolic blood pressure, yet it was not sufficient to induce diastolic dysfunction determined by EDPVR measurements (Supplemental Table 3). OTII mice also had increased renal CD4^+^ T cell abundance in response to H/L compared with STD controls (Supplemental Figure 3, D and E). These data demonstrate that endogenous antigen specificity is dispensable for cardiac and kidney T cell infiltration induced by H/L, and that alternative T cell activation mechanisms are required for diastolic dysfunction.

### H/L imprints a possibly unique signature in CD4^+^ T cells characterized by defective UPR activation.

To investigate alternative mechanisms of CD4^+^ T cell activation taking place in response to systemic metabolic and mechanical stress, we next focused on the UPR. Downregulation of myocardial spliced *Xbp1* (*Xbp1s*) is a hallmark of HFpEF (14), and T cell deficiency of *Xbp1* results in highly inflammatory T cells with enhanced antitumor activity (15). As expected, the expression of myocardial *Xbp1s* was reduced in mice subjected to 5 weeks of H/L, compared with STD and TAC mice, which presented with reduced ejection fraction, LV hypertrophy, and thickening of both the anterior and posterior walls (Supplemental Figure 3, F–I, and Supplemental Table 4), reinforcing that downregulation of myocardial UPR is HFpEF specific. This 5-week time point coincides with systemic expansion of effector T cells in the lymph nodes and the spleen (Figure 2). Total splenic CD4^+^ T cells isolated from H/L mice at this time point manifested a striking downregulation of not only *Xbp1s*, but also total *Xbp1*, activating transcription factor 6 (*Atf6*), and activating transcription factor 4 (*Atf4*). This global UPR downregulation was not observed in splenic CD4^+^ T cells from mice undergoing TAC (Figure 4, E–H) nor in mice single-treated with either l-NAME or HFD only, in which we did not observe total *Xbp1*, *Atf4*, or *Atf6* downregulation, whereas *Xbp1s* was downregulated, but to a significantly lesser extent than with the combination of both stressors, which synergistically decreased T cell *Xbp1s* expression (Supplemental Figure 3J). These data identify a molecular signature specific to cardiometabolic HFpEF in splenic CD4^+^ T cells.

We next assessed whether this signature was observed in circulating T cells, hypothesizing it could be used as a noninvasive biomarker for HFpEF. Whereas downregulation of *Atf4* was also observed in circulating CD3^+^ T cells from H/L mice, unlike in splenic T cells, circulating CD3^+^ T cells from H/L and STD mice manifested comparable expression of *Xbp1s*, total *Xbp1*, and *Atf6*. Moreover, the expression of *Xbp1s*, total *Xbp1*, *Atf6*, and *Atf4* remained unchanged in circulating CD3^+^ T cells from sham and TAC mice, demonstrating that downregulation of circulating T cell *Atf4* is HFpEF specific (Figure 4, I–L). In contrast, no changes in the expression of any of these UPR genes were observed in total peripheral blood mononuclear cells (PBMCs) between H/L-fed mice and STD-fed controls (Supplemental Figure 3K). Interestingly, after 5 weeks of H/L, splenic CD8^+^ T cells from H/L-fed mice also manifested significantly decreased expression of *Xbp1s*, but a complete preservation of *Atf4* (Supplemental Figure 3L). Furthermore, splenic CD4^+^ T cells from female WT mice given H/L did not have significant downregulation of spliced or total *Xbp1* (Supplemental Figure 3M). Taken together, these data demonstrate a downregulation of all 3 branches of the splenic CD4^+^ T cell UPR of mice with cardiometabolic HFpEF, but not mice with HFrEF (TAC). Alterations in *Atf4* gene expression are observed in circulating T cells, but not total PBMCs, in response to H/L, supporting T cell–specific dysregulation of the UPR in HFpEF. Moreover, these data identify sex differences in *Xbp1s* downregulation induced by H/L, as well as T cell type differences in the global transcriptional downregulation of all 3 arms of the UPR, and highlight a possible unique signature in CD4^+^ T helper cells.

### H/L causes temporal dysregulation of the IRE1α-XBP1 arm of the UPR in T cells.

**Figure 5:**
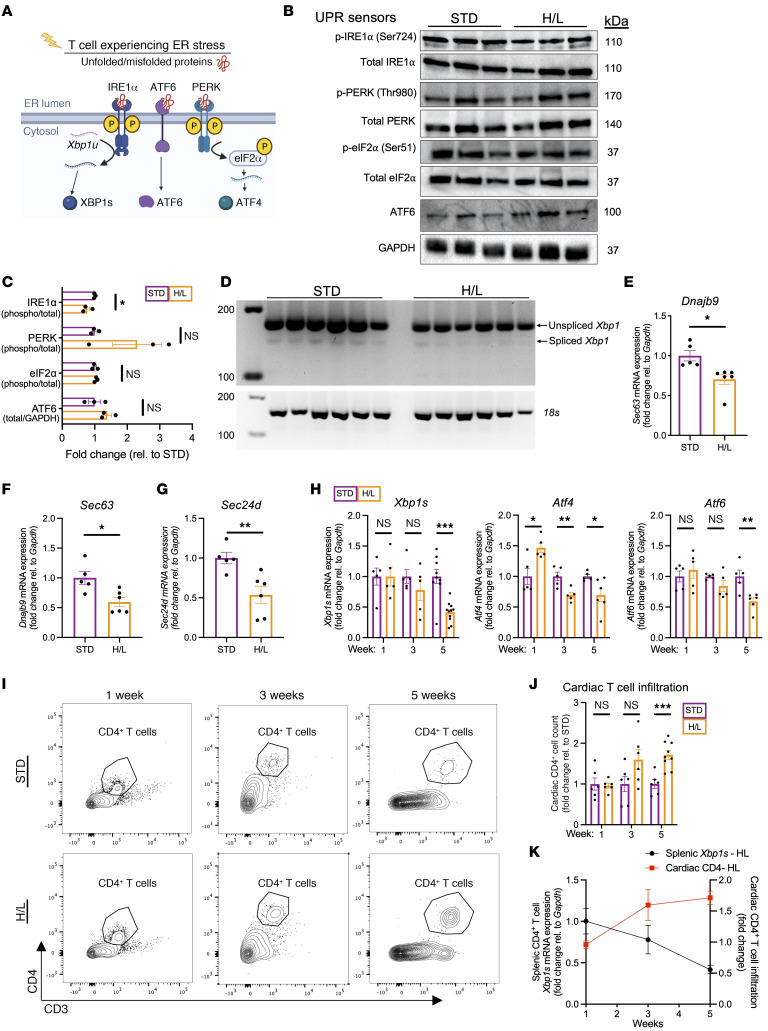
H/L induces dysregulation of T cell UPR gene expression. (**A**) UPR activity can be measured by the gene and protein expression of sensors in the ER lumen and downstream effector proteins. (**B** and **C**) The protein expression and activation of each UPR sensor was analyzed by Western blotting in splenic CD4^+^ T cells from mice fed H/L or STD for 5 weeks. (**D**) Spliced and unspliced *Xbp1* expression was analyzed using semiquantitative PCR and horizontal gel electrophoresis in splenic CD4^+^ T cells from mice fed H/L or STD for 5 weeks. (**E**–**G**) The expression of the XBP1s gene targets *Dnajb9*, *Sec63*, and *Sec24d* was analyzed by quantitative PCR (qPCR) in splenic CD4^+^ T cells from mice fed H/L or STD for 5 weeks. (**H**–**J**) The gene expression of *Xbp1s*, *Atf6*, and *Atf4* was analyzed by qPCR in splenic CD4^+^ T cells from mice fed H/L or STD for 1, 3, or 5 weeks (**H**), and cardiac CD45^+^CD3^+^CD4^+^ cells were analyzed by flow cytometry (**I** and **J**). (**K**) Qualitative representation of splenic CD4^+^ T cell *Xbp1s* expression and cardiac CD4^+^ T cell infiltration in mice fed H/L demonstrates an inverse correlation. **B** and **C**: Each replicate is *n* = 4 mice pooled, and molecular weights are listed next to blots. **C**: The normalized expression of each protein is represented as a fold change relative to STD control. **D**–**J**: *n* = 5–11. Error bars represent the mean ± SEM. **C** and **E**–**G**: Unpaired, 2-tailed *t* test; **H**–**J**: H/L group from each time point is compared against its respective STD control in unpaired, 2-tailed *t* test, and 1- and 3-week time points are plotted against 5-week data from Figure 1 and Figure 4. *P ≤ 0.05, **P ≤ 0.01, ***P ≤ 0.001. This figure was created using Biorender.com.

To further investigate how the T cell UPR in HFpEF is modulated, we next focused on the activation status of the 3 sensors of unfolded and misfolded proteins in the ER that govern the UPR: inositol-requiring enzyme-1α (IRE1α), PKR-like ER kinase (PERK), and activating transcription factor 6 (ATF6). Under ER stress, IRE1α is phosphorylated and splices *Xbp1* to generate *Xbp1s*, which encodes the transcription factor XBP1s. PERK activation leads to ATF4 expression, whereas ATF6 is cleaved and translocates to the nucleus to act as a transcription factor (Figure 5A). Because we observed downregulated expression of *Xbp1s*, *Atf4*, and *Atf6* in CD4^+^ T cells after 5 weeks of H/L (Figure 4, E–H), we next investigated the upstream activation states of IRE1α, PERK, and ATF6. We found that splenic CD4^+^ T cells isolated from WT mice 5 weeks after H/L manifested decreased IRE1α phosphorylation compared with STD-fed mice. In contrast, the phosphorylation of PERK and its downstream target eukaryotic translation initiation factor 2α (eIF2α), as well as ATF6 protein expression, was not significantly decreased (Figure 5, B and C). At the time of impaired T cell phospho-IRE1α expression in response to H/L, we observed defective *Xbp1* splicing in T cells from H/L- compared with STD-fed controls (Figure 5D). Accordingly, the expression of the downstream transcriptional targets of XBP1s, namely DnaJ heat shock protein family member B9 (*Dnajb9*), *Sec63*, and SEC2D homolog D (*Sec24d*), was also decreased in splenic CD4^+^ T cells from H/L mice compared with STD control mice (Figure 5, E–G). Investigation of the temporal dynamics of the UPR effector gene expression in splenic CD4^+^ T cells side by side with analysis of cardiac CD4^+^ T cell infiltration demonstrated that as early as 1 week after H/L, before the onset of cardiac T cell infiltration, T cells responded to ER stress by upregulating *Atf4* expression, with no significant changes in the expression of *Xbp1s* or *Atf6* (Figure 5H). After 3 weeks of H/L, only *Atf4* was significantly downregulated in splenic CD4^+^ T cells, culminating in a potent downregulation of all 3 UPR genes alongside significant cardiac CD4^+^ T cell infiltration after 5 weeks of H/L (Figure 5, H–J). The temporal downregulation of *Xbp1s* in splenic T cells correlated with the sequential cardiac CD4^+^ T cell infiltration observed over time after H/L (Figure 5K).

Taken together, these data indicate that IRE1α is the dominant target of H/L when diastolic dysfunction, CD4^+^ T cell activation, and T cell cardiotropism occur at 5 weeks. Our results also show that as early as 1 week after H/L, before T cell cardiac infiltration, splenic T cells respond to ER stress by upregulating *Atf4*, which, together with other UPR effectors, becomes downregulated over time as T cell cardiotropism and diastolic dysfunction progress in experimental HFpEF.

### XBP1s deficiency improves CD4^+^ T cell persistence in vivo.

**Figure 6:**
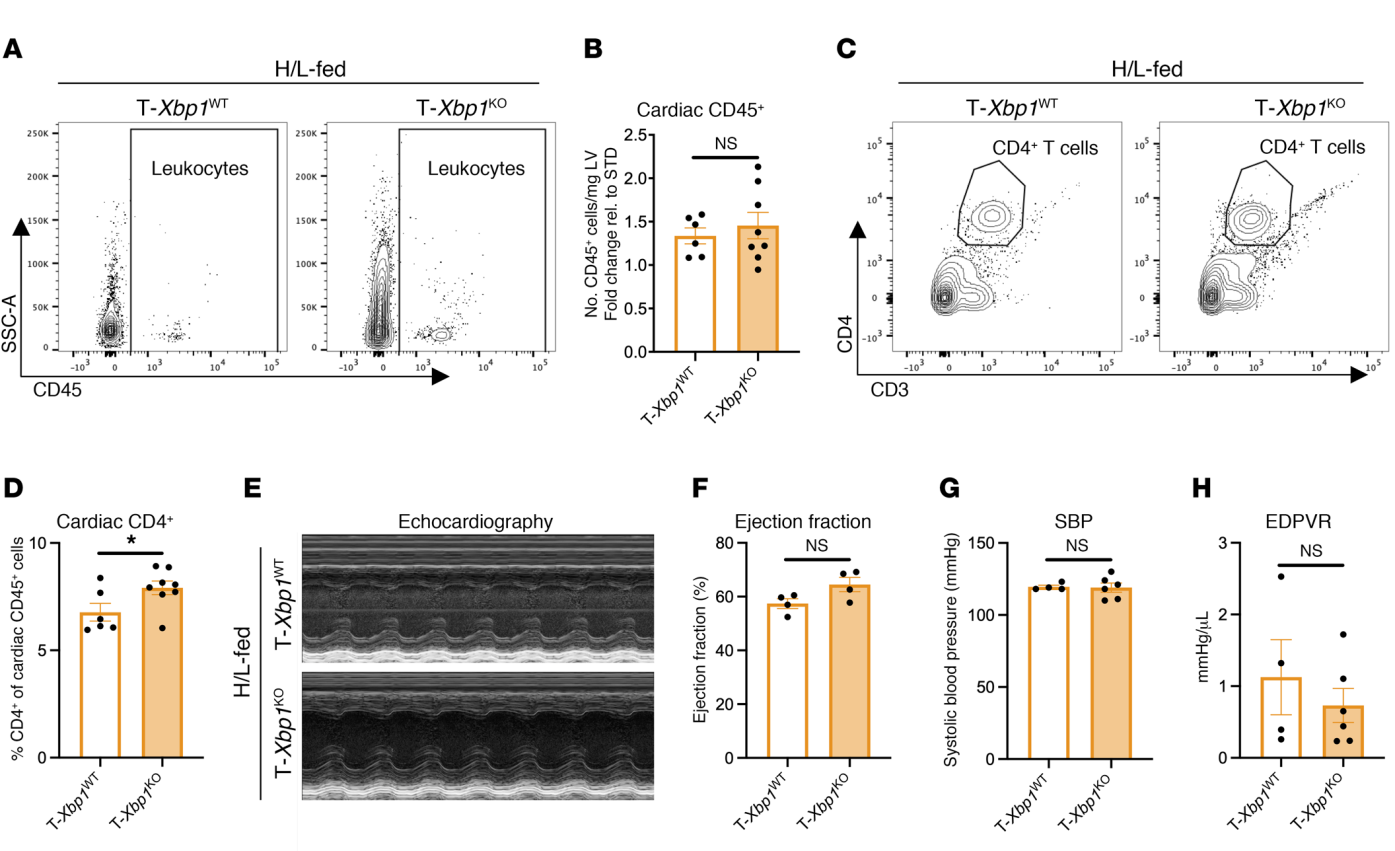
T cell *Xbp1* deficiency increases cardiac CD4^+^ T cell infiltration. CD4^Cre^-*Xbp1^flox^* (T-*Xbp1^KO^*) and CD4^WT^-*Xbp1^flox^* (T-*Xbp1^WT^*) mice were fed H/L for 5 weeks. (**A**–**D**) Cardiac CD45^+^ (**A** and **B**) and CD45^+^CD3^+^CD4^+^ (**C** and **D**) cells were analyzed in LV directly by flow cytometry. (**E**–**H**) Ejection fraction (**E** and **F**) by echocardiography and systolic blood pressure (SBP; **G**) and end-diastolic pressure–volume relationship (EDPVR; **H**) by invasive hemodynamic analysis were measured in each group. Error bars represent mean ± SEM. *n* = 4–8. Unpaired, 2-tailed *t* test. *P ≤ 0.05. This figure was created using Biorender.com.

To assess the functional consequences of decreased XBP1 expression in CD4^+^ T cells in the context of cardiometabolic HFpEF, we next generated CD4^Cre^-*Xbp1^flox^* (T-*Xbp1^KO^*) mice in which all CD4^+^ cells constitutively lack *Xbp1*. We corroborated specific silencing of the gene coding for *Xbp1* in CD4^+^ T cells at the genome level, and the inability to activate XBP1s protein expression by the ER stressor tunicamycin in T cells from T-*Xbp1^KO^* mice (Supplemental Figure 4, A–C). STD-fed T-*Xbp1^KO^* mice manifested no detectable systemic inflammation and no alterations to lymphoid CD4^+^ T cell populations compared with CD4^WT^-*Xbp1^flox^* (T-*Xbp1^WT^*) mice, as previously reported (15). T-*Xbp1^WT^* and T-*Xbp1^KO^* mice fed H/L manifested comparable total cardiac CD45^+^ leukocyte infiltration (Figure 6, A and B). However, we noted a higher frequency of CD45^+^CD4^+^ T cells infiltrating the heart in T-*Xbp1^KO^* animals fed H/L, compared with T-*Xbp1^WT^* control mice, demonstrating increased T cell cardiotropism and suggesting that genetic deletion of *Xbp1* in CD4^+^ T cells improves intracardiac T cell persistence in response to H/L (Figure 6, C and D, and Supplemental Figure 4D). Despite these changes in cardiac T cell abundance, T-*Xbp1^KO^* animals fed H/L manifested similar ejection fraction, systolic blood pressure, and EDPVR compared with their WT littermate controls fed H/L (Figure 6, E–H, and Supplemental Table 5).

**Figure 7:**
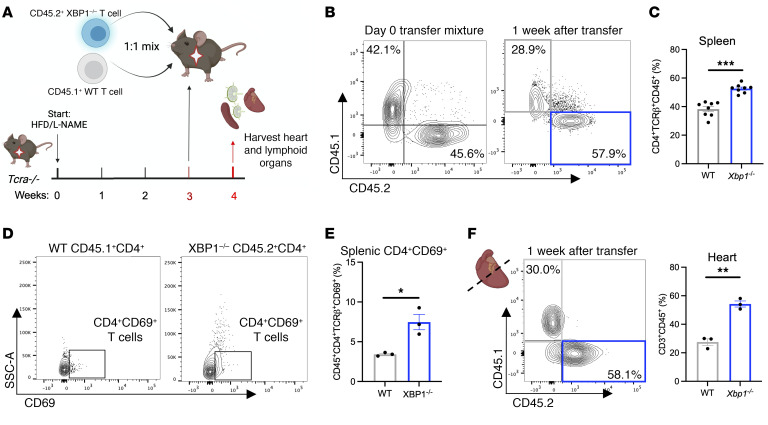
*Xbp1* deficiency improves CD4^+^ T cell persistence in vivo. Splenic CD4^+^ T cells from CD45.2^+^ T-*Xbp1^KO^* mice and CD45.1^+^ WT mice were isolated and expanded in culture into T cell blasts in the presence of αCD3/αCD28 blocking antibodies. (**A**) These cells were injected i.p. in a 1:1 mixture into *Tcra^–/–^* recipients fed H/L for 3 weeks, and spleen, mediastinal and inguinal lymph nodes, and LV were analyzed 1 week after transfer. (**B**–**F**) Relative proportion of each cell population in the spleen (CD4^+^TCRβ^+^CD45.1^+^ or CD45.2^+^, **B** and **C**), expression of CD69 within each cell population in the spleen (**D** and **E**), and relative proportion of each cell population in the LV (**F**) were measured directly by flow cytometry. Error bars represent the mean ± SEM. *n* = 3–8. Unpaired, 2-tailed *t* test. *P ≤ 0.05, **P ≤ 0.01, ***P ≤ 0.001. This figure was created using Biorender.com.

To corroborate our observations that XBP1 deletion increased cardiac T cell infiltration, we performed competitive adoptive cotransfer experiments of a 1:1 ratio of *Xbp1^KO^*CD45.2^+^CD4^+^ to *Xbp1^WT^*CD45.1^+^CD4^+^ cells in T cell–deficient (*Tcra^–/–^*) host recipients fed H/L for 3 weeks (Figure 7, A and B, and Supplemental Figure 4E). *Xbp1^KO^* CD4^+^ T cells outcompeted WT cells in the spleen, as well as other lymphoid organs (mediastinal and inguinal lymph nodes), after only 1 week (Figure 7C and Supplemental Figure 4, F and G), demonstrating that T cell *Xbp1* deficiency imparts a survival advantage in the setting of H/L. We observed a greater increase in expression of CD69, a canonical T cell activation marker, in *Xbp1^KO^* CD4^+^ T cells compared with *Xbp1^WT^* CD4^+^ T cells (Figure 7, D and E). Strikingly, while both *Xbp1^WT^* and *Xbp1^KO^* CD4^+^ T cell populations were able to traffic into the heart of *Tcra^–/–^* recipients subjected to H/L, there was a greater relative abundance of *Xbp1^KO^* CD4^+^ T cells in the heart (Figure 7F and Supplemental Figure 4H). Thus, XBP1 deficiency provides a survival advantage in peripheral organs and results in enhanced cardiotropism in the onset of H/L.

Taken together, these data indicate that lack of *Xbp1* endows T cells with a survival advantage and enhanced cardiotropism, yet it does not further worsen the already decreased diastolic function induced by H/L in experimental cardiometabolic HFpEF.

### Withdrawal of HFD and l-NAME restores T cell IRE1α-XBP1s activation and partially improves cardiac inflammation, hypertrophy, and diastolic dysfunction in cardiometabolic HFpEF.

**Figure 8:**
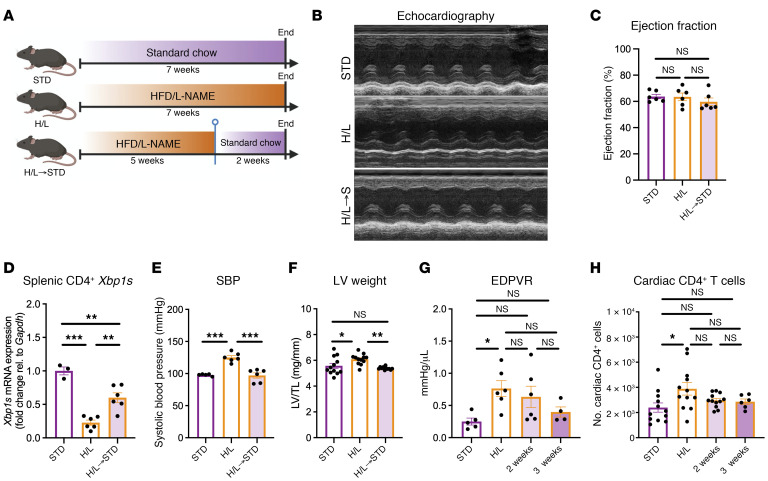
H/L-induced CD4^+^ T cell UPR dysregulation is dependent on the microenvironment. WT mice were fed STD, H/L, or H/L for 5 weeks and then STD for 2 weeks (H/L->S) prior to analysis (**A**). (**B** and **C**) Ejection fraction in these mice was measured by echocardiography. (**D** and **E**) Splenic CD4^+^ T cells were isolated from mice in each group and measured for *Xbp1s* expression by qPCR (**D**), and systolic blood pressure (SBP; **E**) was measured by invasive hemodynamic analysis. (**F**) LV from mice in each group was weighed and normalized to tibia length (LV/TL). (**G** and **H**) In mice from these groups, in addition to a cohort of mice fed H/L for 5 weeks then reverted to STD for 3 weeks, end-diastolic pressure–volume relationship (EDPVR) was quantified by invasive hemodynamic analysis (**G**), and cardiac CD45^+^CD3^+^CD4^+^ cells were analyzed in LV of mice from each group directly by flow cytometry (**H**). *n* = 3–12. Error bars represent the mean ± SEM. One-way ANOVA with Tukey’s multiple-comparison test. *P ≤ 0.05, **P ≤ 0.01, ***P ≤ 0.001. This figure was created using Biorender.com.

Our data revealed a temporal downregulation of the T cell UPR elicited by H/L accompanied by T cell cardiotropism and diastolic dysfunction, and showed that T cell germline deficiency of XBP1 is sufficient to enhance T cell cardiotropism induced by H/L but does not further induce diastolic dysfunction. These findings prompted us to test whether removal of the 2 stressors (HFD and l-NAME) could reverse such T cell signature and have consequences for cardiac inflammation and pathology in cardiometabolic HFpEF. We found that splenic CD4^+^ T cells isolated from mice fed for 5 weeks with H/L or STD recovered the expression of *Xbp1s*, total *Xbp1*, *Atf6*, and *Atf4* upon incubation for 4 hours in complete media lacking HFD and l-NAME (Supplemental Figure 5, A–E), demonstrating that defective T cell UPR activation is transient and conditional on the presence of a metabolically challenging microenvironment in vitro. We next tested the hypothesis that withdrawal of the 2 hits of cardiometabolic HFpEF restores CD4^+^ T cell *Xbp1s* expression in vivo and impacts cardiac inflammation and function. After 5 weeks of H/L feeding, sufficient to induce HFpEF-like pathology and splenic CD4^+^ T cell UPR downregulation, mice were fed STD diet for 2 weeks and compared with controls fed STD or mice fed H/L for the duration of the study (Figure 8A). All groups maintained their ejection fraction throughout the study (Figure 8, B and C, and Supplemental Table 6). Reversion to STD for 2 weeks was sufficient to partly recover the expression of *Xbp1s* in total splenic CD4^+^ T cells (Figure 8D) and resulted in a nearly 30 mmHg decrease in systolic blood pressure and decrease in LV weight in comparison with mice maintained on H/L (Figure 8, E and F). These results, together with the data demonstrating a temporal splenic CD4^+^ T cell UPR response that correlates with cardiac CD4^+^ T cell infiltration in the onset of H/L, at time points that precede onset of preclinical HFpEF (Figure 5, H–J), led us to next extend the time course of H/L withdrawal to 3 weeks on STD following 5 weeks of H/L, to further test whether cardiac inflammation and diastolic dysfunction lag behind the splenic CD4^+^ T cell UPR expression. We found that both 2 and 3 weeks of STD after 5 weeks of H/L resulted in a downward trend, but were insufficient to fully recover cardiac diastolic function (Figure 8G) or cardiac CD4^+^ T cell infiltration (Figure 8H and Supplemental Figure 5F).

Taken together, these data demonstrate that the splenic expression of CD4^+^ T cell *Xbp1s* is recovered by removal of the 2 hits of HFpEF in vitro and in vivo, and results in ameliorated CD4^+^ T cell cardiac infiltration and cardiac hypertrophy as well as partial improvement in diastolic dysfunction. Removal of the 2 hits results in immediate recovery of CD4^+^ T cell *Xbp1s*, whereas the full recovery of cardiac diastolic function and T cell infiltration lags behind this early response in the UPR.

## Discussion

In this study, we present experimental evidence indicating that diastolic dysfunction and cardiomyocyte hypertrophy are T cell dependent in the 2-hit preclinical model of cardiometabolic HFpEF that combines the most common HFpEF risk factors: hypertension and obesity. We identify an HFpEF T cell response that is distinct from HFrEF and does not require cardiac antigen recognition when diastolic dysfunction initiates. Instead, the HFpEF T cell response is characterized by what we believe is a novel molecular T cell signature featuring impaired IRE1α-XBP1s signaling that promotes T cell cardiotropism, intracardiac persistence, and inflammatory potential. Moreover, we discovered that withdrawal of the 2 hits that induce this response partially restores T cell *Xbp1s* expression and ameliorates cardiac inflammation, cardiomyocyte hypertrophy, and diastolic dysfunction.

Our data in wild-type mice uncover that CD4^+^ effector T cells are expanded in the lymphoid organs close and distal to the heart and infiltrate the heart shortly after mice are fed H/L in experimental cardiometabolic HFpEF, coexisting with cardiac hypertrophy and diastolic dysfunction. Whereas there is also cardiac infiltration of myeloid cells and B cells, our data in *Tcra^–/–^* mice demonstrate a causal role of T cells in cardiomyocyte hypertrophy and diastolic dysfunction. Previous studies by our group and others have reported the necessity of T cells to develop cardiac and vascular inflammation in HFrEF and hypertension, respectively, using a variety of T cell–deficient mice (22–24). Our studies herein demonstrate a central role for T cells in diastolic dysfunction and the associated remodeling in the context of similar increases of systolic blood pressure in *Tcra^–/–^* and WT mice subjected to H/L. Furthermore, our observations of enhanced proinflammatory cytokine secretion in response to H/L corroborate recent preclinical studies identifying enhanced T cell cytokine production, including IFN-γ, in the hearts of mice with HFpEF induced by deoxycorticosterone acetate/salt hypertension (25). Mechanistically, we found that H/L downregulates cardiac phospho-phospholamban expression in a T cell–dependent manner, which ostensibly contributes to diastolic dysfunction through increased inhibition of SERCA activity in WT mice, and affords protection to *Tcra^–/–^* mice. Our team has previously published that H/L decreases myocardial *Xbp1s*, and that overexpression is sufficient to revert experimental HFpEF in a T cell–sufficient environment, supporting that cardiomyocyte XBP1s is ultimately important for mediating cardiac relaxation. Our data with T cell deficiency reveal what we believe to be a new mechanism, and underscore the importance of studying T cell–cardiomyocyte interactions in preclinical HFpEF, and how molecular alterations of key mediators in each cell type affect disease progression. Phospholamban may be phosphorylated by cAMP-dependent protein kinase (PKA) following stimulation of β-adrenergic receptors (20) or by cGMP-dependent protein kinase G Iα (PKGIα) signaling (26). PKGIα signaling has been shown to directly modulate the EDPVR in preclinical settings (26), and decreased myocardial PKG activity has been shown in patients with HFpEF, but not those with HFrEF (27). Thus, it is possible that T cells modulate these axes, directly through soluble factor secretion or indirectly through cell-cell interactions with stromal cells that act on these axes specifically in the context of HFpEF; these possibilities are ongoing areas of investigation.

Importantly, the 2 hits inducing metabolic and mechanical stress are simultaneously required to induce T cell lymphoid activation, cardiac infiltration, and T cell–mediated cardiac remodeling. Moreover, unlike in HFrEF and hypertension, in which the mechanism of T cell activation involves endogenous antigen recognition in the heart and the vasculature, respectively (7, 11, 28), our studies using Nur77^GFP^ and OTII mice demonstrate this is not the case in HFpEF. Indeed, CD4^+^ T cells in OTII mice become activated by H/L and infiltrate the heart in the absence of their cognate antigen, ovalbumin, further supporting that systemic perturbations activate CD4^+^ T cells in HFpEF in an endogenous antigen-independent manner. Thus, our data differentiate T cell inflammation in HFpEF from HFrEF by the lack of cardiac antigen specificity and directly contrast T cells in HFpEF and HFrEF by their UPR expression. We speculate that the difference in how T cells mediate systolic and diastolic function in HFpEF versus HFrEF is likely due to the differences in T cell responses and phenotypes that directly impact T cell–cardiomyocyte crosstalk in each of these disease contexts. Furthermore, our observations that T cells infiltrate the heart in an antigen-independent manner in preclinical HFpEF align with our data in WT mice showing that effector T cell expansion is not limited to the cardiac draining lymph nodes and is evident in systemic homing organs such as the spleen, as well as our data demonstrating increased renal CD4^+^ T cell infiltration in both WT and OTII mice fed H/L. Though we observe increased renal and cardiac T cell infiltration following 5 weeks of H/L feeding in WT mice, we did not observe changes in plasma blood urea nitrogen (BUN) concentrations, suggesting that T cell contributions to cardiac pathology occur earlier than possible contributions to renal dysfunction in preclinical HFpEF. This degree of organ specificity may be due to the essential function of myocytes, which, owing to active T cell–cardiomyocyte crosstalk, are perhaps more sensitive to T cell inflammation than kidney cells. Studies investigating longer time points of H/L treatment will interrogate whether T cell–mediated renal pathology occurs in chronic stages of cardiometabolic HFpEF, as is observed in patients (3). Furthermore, the inability of OTII CD4^+^ T cells to induce diastolic dysfunction, despite the increase in systolic blood pressure, suggests that additional mechanisms are involved in diastolic dysfunction in HFpEF.

Our studies demonstrate a possible unique T cell signature induced exclusively by the combination of H/L, and not in experimental HFrEF induced by TAC: IRE1α-XBP1 downregulation in splenic CD4^+^ T cells. Decreased myocardial expression of *Xbp1s* is a hallmark of HFpEF and is mediated in cardiomyocytes by nitrosylation of IRE1α, which, in turn, prevents IRE1α phosphorylation (14). We show that the same axis is impaired in CD4^+^ T cells and additionally observe CD4^+^ T cell downregulation of total *Xbp1*, *Atf6*, and *Atf4* expression, demonstrating global downregulation of transcripts encoding key UPR transcription factors in response to H/L. CD8^+^ T cells, which infiltrate the heart in response to H/L although to a lesser extent than CD4^+^ T cells, also manifested downregulation of *Xbp1s*, but not other arms of the UPR, which were largely preserved. Previous work, including that published by our groups, has characterized the role of UPR modulation in dendritic cells (29), myeloid-derived suppressor cells (30), and CD8^+^ T cells (16), all in the context of metabolically challenging tumor microenvironments. These studies highlight cell-specific mechanisms by which the UPR impacts cell fate. A common theme among these studies is the finding that impaired UPR signaling increases the inflammatory potential of each of these cell types, and our observations complement this paradigm. Additionally, our data demonstrate that female mice are protected in this model of preclinical HFpEF, in accordance with previous studies by our group (18), despite having similar increases in cardiac infiltrating CD4^+^ T cells, yet CD4^+^ T cells in females subjected to H/L did not exhibit downregulation of *Xbp1*. It is possible that the dynamics of cardiomyocyte phospholamban activity differ in female mice and that CD4^+^ T cell XBP1, unaltered in females, contribute to this response, potentially as it relates to β-adrenergic and/or PKG1α signaling and T cell–cardiomyocyte crosstalk in cardiometabolic HFpEF. These sex-specific differences merit further investigation as we continue learning about the role of inflammation in HFpEF.

The concept that metabolic perturbations lead to alterations in the T cell UPR response has been described previously in other contexts of metabolically challenging environments, such as the tumor microenvironment. Indeed, dysregulated UPR activation in intratumoral immune cells results in altered proinflammatory activity, T cell dysfunction, and tumor immune evasion (16, 30–33). Furthermore, deletion of *Xbp1* has been shown to enhance T cell effector function in the ovarian cancer microenvironment (15). Our results in WT mice subjected to H/L, together with our data using CD4^Cre^-*Xbp1^fl/fl^* mice, corroborate this phenomenon in a different disease setting and support the paradigm that the impaired IRE1α activation and reduced XBP1s expression in an unfavorable milieu shift T cells toward a more inflammatory phenotype that includes elevated expression of IFN-γ and IL-4, enhanced survival in the H/L microenvironment, and prolonged contributions to adverse cardiac remodeling in experimental cardiometabolic HFpEF, in a way that is not restricted to cardiac antigens. This T cell signature adds to our knowledge of how molecular differences between HFpEF and HFrEF, recently identified in patients at the myocardial metabolomic level (34), may shape T cell immunity and cardiac inflammation. Additionally, given that obesity is a risk factor for many cancers, dysregulation of the T cell UPR may represent a shared disease mechanism in these 2 diseases.

Our results analyzing T cell UPR genes over time after H/L also reveal that the T cell regulation of this pathway is modulated over time and is highly dynamic. Moreover, we show that *Xbp1s* downregulation is transient and reversible when the cardio- and metabolic stressors are withdrawn, as shown in mice reverted to STD after 5 weeks of H/L. In these mice, cardiac hypertrophy and systolic blood pressure were reduced in comparison with mice remaining on H/L throughout the duration of the experiment, and these changes were guided by partial reversal of CD4^+^ *Xbp1s* downregulation and subsequent decline in cardiac T cell infiltration. The development of effective treatment strategies for HFpEF has remained an extraordinary clinical challenge, and, to date, only empagliflozin has provided improvements in HFpEF patient quality of life in comparison with placebo-treated patients in clinical trials (35). Lifestyle interventions, such as changes in diet and exercise, have been shown to be effective in ameliorating symptoms of HF and improving cardiac function in HFpEF patients (36–38). It will be interesting to investigate whether changes in systemic T cell function through *Xbp1* regulation contribute to these beneficial effects. Furthermore, the effects of SGLT2 inhibition and exercise on the T cell UPR may prove informative.

Our reversal studies showing that T cells respond to the altered environment and recover the UPR gene expression, alongside the observed recovery of LV hypertrophy, may reveal a new way to monitor patient health in response to lifestyle interventions, using T cell UPR fluctuations as a potential indicator for disease progression or regression. However, recovery of diastolic function and cardiac T cell abundance was not achieved when we extended the time course to recover mice on STD for 3 weeks after 5 weeks of H/L, indicating that these responses lag behind the CD4^+^ T cell UPR expression. Additional studies will interrogate whether longer withdrawal is necessary to recover these pathologies, or whether they are irreparable.

There is also a great need for molecular biomarkers of HFpEF that may assist in diagnosis, disease management, and characterization of disease progression. Whereas our results demonstrate a distinct UPR signature in splenic T cells, circulating PBMCs, which would be an ideal source as a biomarker, do not show such a signature. This finding is in line with recent data demonstrating that HFpEF, HFrEF, and healthy patients have distinct metabolomic signatures in tissues, but these differences are lost in the plasma (34) — thus, the differences in tissue and circulating microenvironments in cardiometabolic HFpEF may drive the emergence and loss of T cell *Xbp1* dysregulation that we observe in the tissue and plasma, respectively. Nevertheless, the T cell component of the PBMCs shows significant downregulation of *Atf4* expression compared with circulating T cells from control mice or mice subjected to HF induced by TAC. These data suggest that *Atf4* downregulation is sustained in the circulation, likely regulated through mechanisms different from those that regulate IRE1α-XBP1s, and could be monitored in blood as a potential less invasive biomarker. Comprehensive clinical studies evaluating changes in T cell UPR expression from HFpEF, HFrEF, and non-HF control patients are necessary to determine the informative capacity of this molecular signature as a clinical biomarker.

Despite these interesting findings presented herein, there are some limitations. We report that both CD4^+^ and CD8^+^ T cells are increased in the heart in response to H/L, yet we do not interrogate their relative contribution to diastolic dysfunction and cardiac hypertrophy. *Xbp1s* expression is reduced in both CD4^+^ and CD8^+^ T cells 5 weeks after H/L, although other UPR arms are only altered in CD4^+^ T cells, which are present in greater numbers than CD8^+^ T cells in the heart. In a similar vein, OTII mice are deficient in CD8^+^ cell development; this impairment may contribute to the absence of diastolic dysfunction in these mice, necessitating further study of CD8^+^ T cell–mediated cardiac remodeling in response to H/L. Because we use the CD4^Cre^ driver, XBP1s is deleted in early thymic development in CD4^+^CD8^+^ double-positive cells, resulting in XBP1s deletion in CD8^+^ cells as well. Furthermore, germline deletion of T cell XBP1s did not worsen cardiac pathology in comparison with WT fed H/L, in which T cell *Xbp1s* is already downregulated by the H/L microenvironment. Nevertheless, our results provide evidence of a central role for *Xbp1s* downregulation in T cell survival and proinflammatory activity that has consequences in the pathophysiology of HFpEF.

Whereas we do not directly study whether overexpression of XBP1s in T cells ameliorates HFpEF, our reversal studies withdrawing the 2 hits of HFpEF support the notion that cardiac inflammation is reduced when the expression of *Xbp1* is enhanced under such conditions. Moreover, we report temporal changes in T cell UPR gene expression over time after H/L, yet further studies are needed to determine the directionality of global UPR downregulation and how each of these elements is downregulated by the 2 hits. Whereas nitrosylation of T cell IRE1α may drive downregulation of *Xbp1s*, as observed in the myocardium of H/L-fed mice (14), we observe downregulation of total *Xbp1*, *Atf4*, and *Atf6*, suggesting alterations in others of the several regulatory checkpoints of the UPR (39), which will be explored in future studies. The UPR also transcriptionally targets pro-apoptotic pathways to eliminate aberrant cells (13); future studies will specifically interrogate whether impaired UPR activation alters such pathways to improve T cell survival in the context of HFpEF to expand on our findings of improved CD4^+^ tissue persistence in response to H/L. Our data demonstrate cardiac CD4^+^ T cells expressing high levels of IFN-γ and IL-4, yet how these modulate cardiomyocyte relaxation specifically needs further investigation. Additionally, the well-established 2-hit model of HFpEF mimics only one of the heterogeneous endotypes of HFpEF; whether this T cell signature is altered differently in other preclinical HFpEF models (40–43) is an area of ongoing investigation. Lastly, while recent RNA sequencing approaches have identified enrichment in inflammatory pathways in HFpEF patient myocardium compared with healthy controls (6), it is of paramount importance to corroborate our data presented herein with clinical data to determine whether T cell UPR downregulation is a clinically relevant biomarker in HFpEF patients, and whether it offers diagnostic value.

In summary, results reported here highlight important differences in T cell function in cardiometabolic HFpEF and HFrEF, and reveal a possible novel proinflammatory T cell signature and a mechanism that enhances T cell cardiac inflammation in HFpEF. Furthermore, our study suggests that lifestyle interventions may improve disease progression through alterations that modulate T cell effector function through modulation of the UPR.

## Methods

### Experimental animals.

C57BL/6J mice were used for wild-type studies. In addition, *Tcra^–/–^* (B6.129S2-Tcratm1Mom/J), Nur77^GFP^ [C57BL/6-Tg(Nr4a1-EGFP/cre)820Khog/J], CD45.1 (B6.SJL-Ptprca Pepcb/BoyJ), and CD4^Cre^ [B6.Cg-Tg(Cd4-cre)1Cwi/BfluJ] mice were purchased from The Jackson Laboratory, bred, and maintained in our facilities. *Xbp1^fl/fl^* mice were a gift from Laurie Glimcher (Harvard Medical School and Dana-Farber Cancer Institute, Boston, Massachusetts, USA) and Juan Cubillos-Ruiz (Department of Obstetrics and Gynecology and Sandra and Edward Meyer Cancer Center, Weill Cornell Medicine and Weill Cornell Graduate School of Medical Sciences, New York, New York, USA), and were crossed with CD4^Cre^ mice to generate CD4^Cre^*-Xbp1^fl/fl^* progeny, which were bred and maintained in our facilities. OTII [B6.Cg-Tg(TcraTcrb)425Cbn/J] mice were a gift from Caroline Genco(Tufts University School of Medicine and Tufts University Graduate School of Biomedical Sciences, Boston, Massachusetts, USA) and were consequently bred and maintained in our facilities. Male adult mice, aged 8–12 weeks, were used in experiments. Animals had unrestricted access to food (standard chow [STD], Envigo Teklad 2918 standard chow; high-fat diet [HFD], Research Diet D12492). For groups receiving l-NAME, l-NAME (0.5 g/L; Sigma-Aldrich) was added to acidified drinking water and provided ad libitum. To induce pressure overload, mice received transverse aortic constriction (TAC) through minimally invasive surgery to constrict transverse aorta (27G), as previously reported (7, 9, 10, 22). Sham-operated mice, which received surgery without aortic constriction, were used as controls. Mice were euthanized and tissues harvested after 1, 3, 5, or 7 weeks of respective diets or surgery.

### In vivo echocardiography and hemodynamics.

Live imaging using transthoracic echocardiography was used in mildly sedated mice as previously described (44). Mice were kept on a heated stage in supine position, with heart and respiratory rates continuously monitored via stage electrodes. Depilatory cream (Nair) was used to remove fur on the chest, and ultrasonic gel was applied for measurement by the 22- to 55-MHz echocardiography transducer (MS550D; Vevo 2100, FUJIFILM VisualSonics). Heart rate was kept between 450 and 550 bpm. M-mode and 2-dimensional images were obtained from short axis view. LV parameters were measured by averaging of values obtained from 8 cardiac cycles. For invasive hemodynamic analyses, a pressure-volume transducer catheter (1.0 F; PVR-1045, Millar Instruments) was introduced into LV through the right carotid artery of anesthetized mice. Mice were maintained at 37°C using a rectal thermometer attached to an automatic feedback heat lamp. Using pressure-volume loop measurements, LV function was measured, with volume measurements calibrated using saline injection parallel conductance method as previously described (44). Data were collected and analyzed using IOX software (version 1.10.8.34, EMKA Instruments).

### Flow cytometry.

Changes in immune cell populations in several tissues were assessed using quantitative flow cytometry. LV samples were digested for 30 minutes with collagenase type II (0.895 mg/mL; Gibco, 17101-015) at 37°C. Kidneys were digested for 45 minutes with collagenase type II (200 U/mL; Gibco, 17101-015) at 37°C in DMEM plus 2% FBS. Mediastinal lymph nodes and spleen were crushed through 40 μm filters using FACS buffer (PBS plus 2% FBS). Cell surface staining of cell suspensions was performed with relevant antibodies and Fc block (anti-CD16/CD32 [αCD16/32], BioLegend, clone 93) diluted 1:50 in FACS buffer for 20 minutes in the dark at 4°C, followed by washes with FACS buffer. For staining intracellular proteins in cardiac samples, CD45^+^ cells were sorted out of cardiac suspension using positive selection CD45 microbeads and magnetically assisted cell sorting (MACS) (Miltenyi Biotec, 130-052-301). The resulting fraction was incubated for 4 hours at 37°C with RPMI T cell complete media with 0.1% ionomycin (Sigma-Aldrich, 13909), 0.1% brefeldin A (BioLegend, 420601), 0.1% monensin (BioLegend, 420701), and 50 ng/mL of phorbol myristate acetate (Sigma-Aldrich, P8139) to make intracellular cytokines detectable by flow cytometry. Samples were then washed, stained for extracellular markers as described above, then fixed for 20 minutes at room temperature using Fixation buffer (BioLegend, 420801). After fixation and washing with FACS buffer, samples were permeabilized by washing twice with 1× permeabilization buffer (BioLegend, 421002), then incubated with relevant antibodies in 1× permeabilization buffer for 20 minutes in the dark at 4°C. Samples were then washed with FACS buffer, and resuspended for flow cytometry. Precision Count Beads (BioLegend 424902) were added to samples to quantify absolute cell numbers. Flow cytometry data were acquired on a BD LSRII (BD Biosciences) and FACSDiva software, and analyzed using FlowJo software (BD Biosciences). The antibodies used are listed in Supplemental Table 7.

### Histology.

Murine LV tissue was frozen, embedded in OCT freezing medium, and sectioned using a cryostat at 10 μm thick. For immunohistochemical staining of cardiac CD4^+^ T cells, samples were fixed in cold acetone (Sigma-Aldrich, 179124), then blocked for 10 minutes in 10% normal goat serum (Abcam, 7481), biotin/avidin blocking kit (Vector Laboratories, SP-2001), and 1% hydrogen peroxide (H1009). Primary antibody (anti-CD4, BioLegend, 100402) was added for 1 hour in a 1:200 dilution, followed by incubation with goat anti-rat biotinylated secondary antibody (1:300 dilution; Jackson ImmunoResearch, 112-065-062). Samples were then incubated with the following in succession: streptavidin-HRP (DAKO K0675), AEC substrate (Sigma-Aldrich, A5754), nuclear counterstain (hematoxylin). Sections were then mounted using aqueous Fluoromount G (Southern Biotech, 0100-01), and stained CD4^+^ T cells were counted manually in each section. For wheat germ agglutinin (WGA) staining, sections were thawed, fixed for 15 minutes with 4% paraformaldehyde, washed twice with PBS, then blocked with 10% normal goat serum for 1 hour. Sections were then stained with WGA (5 μg/mL; Sigma-Aldrich, L4895) for 2 hours in a humidified chamber protected from light, followed by 2 washes with PBS and mounting with Fluoromount DAPI (Southern Biotech, 0100-20). Visualization was performed with a Nikon Ti inverted fluorescent microscope.

### Urea nitrogen (BUN) detection assay.

To identify kidney dysfunction, blood urea nitrogen (BUN) concentration was measured using a colorimetric detection kit (EIABUN, Thermo Fisher Scientific). Plasma was isolated from blood by centrifuging of heparinized whole-blood samples at 12,000*g* for 25 minutes at 4°C. Plasma samples were then diluted 1:20 with deionized water, combined with kit reagents per the manufacturer’s instructions, and absorbance was read at 450 nm against a standard curve that was provided with the kit.

### Isolation of splenic and blood T cells for culture and RNA isolation.

Total splenic CD4^+^ or CD8^+^ T cells were sorted out of the spleen using positive selection CD4 (Miltenyi Biotec, 130-117-043) or CD8 microbeads (Miltenyi Biotec, 130-117-044) for MACS. For isolation of circulating leukocytes/T cells, murine blood was collected in a heparinized needle, then lysed using a red blood cell lysis kit (BioLegend, 420302). Resulting CD45^+^ fraction was either used for RNA extraction of PBMCs, or MACS-sorted for CD3^+^ T cells using Pan T Cell isolation kit (Miltenyi Biotec, 130-095-130). For CD3^+^ T cell, PBMC, and CD8^+^ T cell isolation, mice were pooled in duplicate to ensure a sufficient quantity of cells for analysis.

For generation of T cell blasts, isolated splenic CD4^+^ T cells were plated at 2 million cells/mL in complete media and differentiated in the presence of αCD3 (2.5 μg/mL; BioLegend, 100253) and αCD28 (1 μg/mL; BioLegend, 102102) for 3 days at 37°C, then harvested for adoptive transfer experiments. T cell complete media consisted of RPMI medium (Gibco, 11875-093) with the following supplements: 10% FBS (Atlanta Biologicals, S11150), Glutamax (Gibco, A12860-01), NaHCO_3_ (Gibco, 25080-094), penicillin/streptomycin (Gibco, 15140122), 60 μM β-mercaptoethanol (Sigma-Aldrich, 444203), and sodium pyruvate (Gibco, 11360-070).

### Quantitative PCR, splicing assay, and Western blotting.

Total RNA was extracted from murine LV using TRIzol (Qiagen, 79306) and consequent chloroform extraction with centrifugation to generate an aqueous phase containing RNA. Aqueous phase was then processed using Qiagen RNeasy Mini Isolation Kit (Qiagen, 74004). Splenic or blood T cells were isolated as described above, then lysed in RLT buffer (Qiagen), and RNA was isolated using Qiagen RNeasy Mini Isolation Kit. RNA was assessed for quality and concentration using Nanodrop One (Thermo Fisher Scientific), then reverse-transcribed using a high-capacity cDNA reverse transcription kit (Thermo Fisher Scientific, 4368814). For quantitative PCR (qPCR) analysis, 20 ng/well of cDNA was used for each reaction, in triplicate, with appropriate primers and SYBR master mix (Thermo Fisher Scientific, A25741). qPCR was performed using a QuantStudio 6 Flex Real-Time PCR Machine (Thermo Fisher Scientific). The 2^–ΔΔCt^ relative quantification method, using *Gapdh* for normalization, was used to calculate fold change ratios relative to mRNA expression of negative control samples. The sequences for primers are included in Supplemental Table 8.

For *Xbp1* splicing assay, cDNA from murine splenic CD4^+^ was subjected to semiquantitative PCR using primers included in Supplemental Table 8. Reaction was conducted using Denville Taq Polymerase (Thomas Scientific CB4050-3). Resulting digest was run on 3% regular agarose gel at 90 V.

For Western blotting, isolated splenic CD4^+^ cells from 4 mice from each group pooled (to ensure sufficient quantity of cells) were lysed on ice with RIPA buffer (150 mM NaCl, 50 mM Tris [pH 7.4], 1% NP-40, 0.1% SDS, 5 mM EDTA, 0.1% sodium deoxycholate, 1 mM DTT) containing proteinase inhibitors (Roche, 04693159001) and phosphatase inhibitors (Roche PHOSS-RO), sonicated, and quantified using DC protein assay (Bio-Rad, 5000112). Proteins were separated using SDS-PAGE on 8% or 10% gels, then transferred to PVDF membranes. Blots were blocked for 1 hour with 5% BSA or nonfat milk, in Tris-buffered saline plus 0.1% Tween-20, depending on specifications of individual antibodies. Incubation in primary antibodies diluted 1:1,000 in respective blocking buffer was conducted overnight at 4°C (antibodies listed in Supplemental Table 9). Blots were then incubated with secondary antibody conjugated to HRP (1:2,000; anti-rabbit, Cell Signaling Technology, 7074) at room temperature for 1 hour. ECL or West Atto detection reagents were used (Thermo Fisher Scientific, 32106 and A38555, respectively) and imaged on a Bio-Rad ChemiDoc MP imager. Band intensity was quantified using ImageJ (NIH).

### Statistics.

Statistical analysis and graphing were performed using GraphPad Prism 9.4.1. All data herein are presented as the mean ± SEM. Analysis was performed using an unpaired, 2-tailed *t* test, 1-way ANOVA with Tukey’s multiple-comparison test, or 2-way ANOVA with Šidák’s multiple-comparison test, as indicated, with differences noted as statistically significant when *P* ≤ 0.05. Grubbs’s test was used to exclude statistical outliers.

### Study approval.

All animal studies were approved by the Tufts University Institutional Animal Care and Use Committee. Mice were bred and maintained under pathogen-free conditions in animal facilities located at Tufts University, and were in compliance with all ethical regulations, as outlined by the *Guide for the Care and Use of Laboratory Animals* (National Academies Press, 2011).

### Data availability.

Underlying data are provided in the Supporting Data Values file. Other researchers may request data, methods, and materials, which will be made available for those aiming to reproduce results or replicate procedures.

## Author contributions

SS designed the project, performed experiments, analyzed data, and wrote the manuscript. ALB performed T cell isolations and edited the manuscript. KK helped with mouse generation (CD4^Cre^-*Xbp1^flox^* generation) and provided intellectual input. ES helped with histological staining of cardiac sections. MA performed all TAC surgeries and hemodynamic analysis. MEF, EBT, and GGS provided intellectual input. JAH aided data interpretation and provided intellectual and technical assistance. RMB analyzed cardiac physiological data, provided intellectual support, and edited the manuscript. JRCR provided technical and conceptual support, aided data interpretation, and edited the manuscript. PA designed the project, provided intellectual support, and edited the manuscript.

## Supplementary Material

Supplemental data

Supporting data values

## Version 1 10/24/2023

In-Press Preview

## Version 2 12/15/2023

Electronic publication
