# Supplementary material for: Impaired T cell IRE1α/XBP1 signaling directs inflammation in experimental heart failure with preserved ejection fraction
Source: J Clin Invest. 2023 Dec 15;133(24):e171874. doi: 10.1172/JCI171874 (PMC10721145; doi:10.1172/JCI171874)
Supplement: Supplemental data [file jci-133-171874-s116.pdf]

Supplementary Figure 1

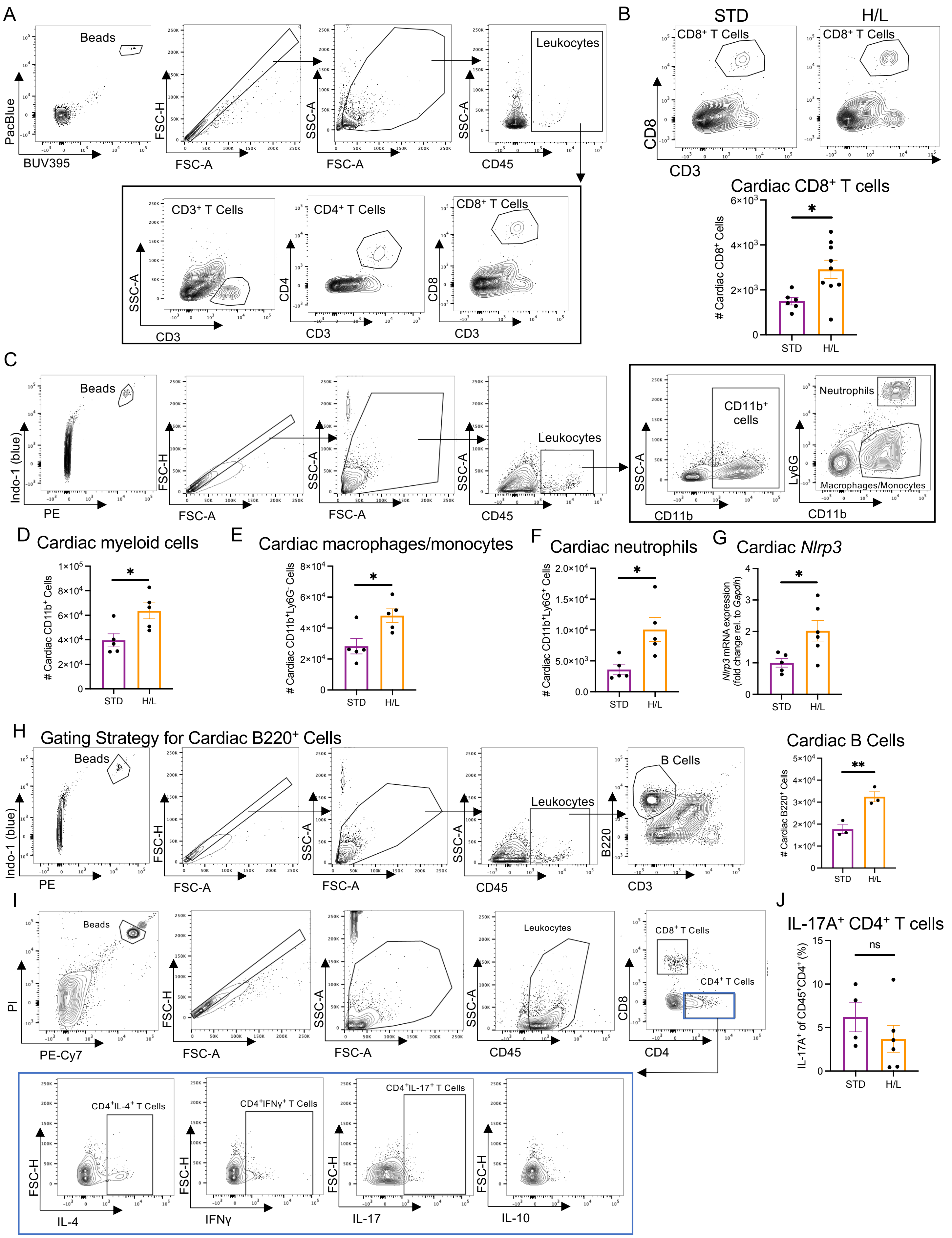

Supplementary Figure 1 continued

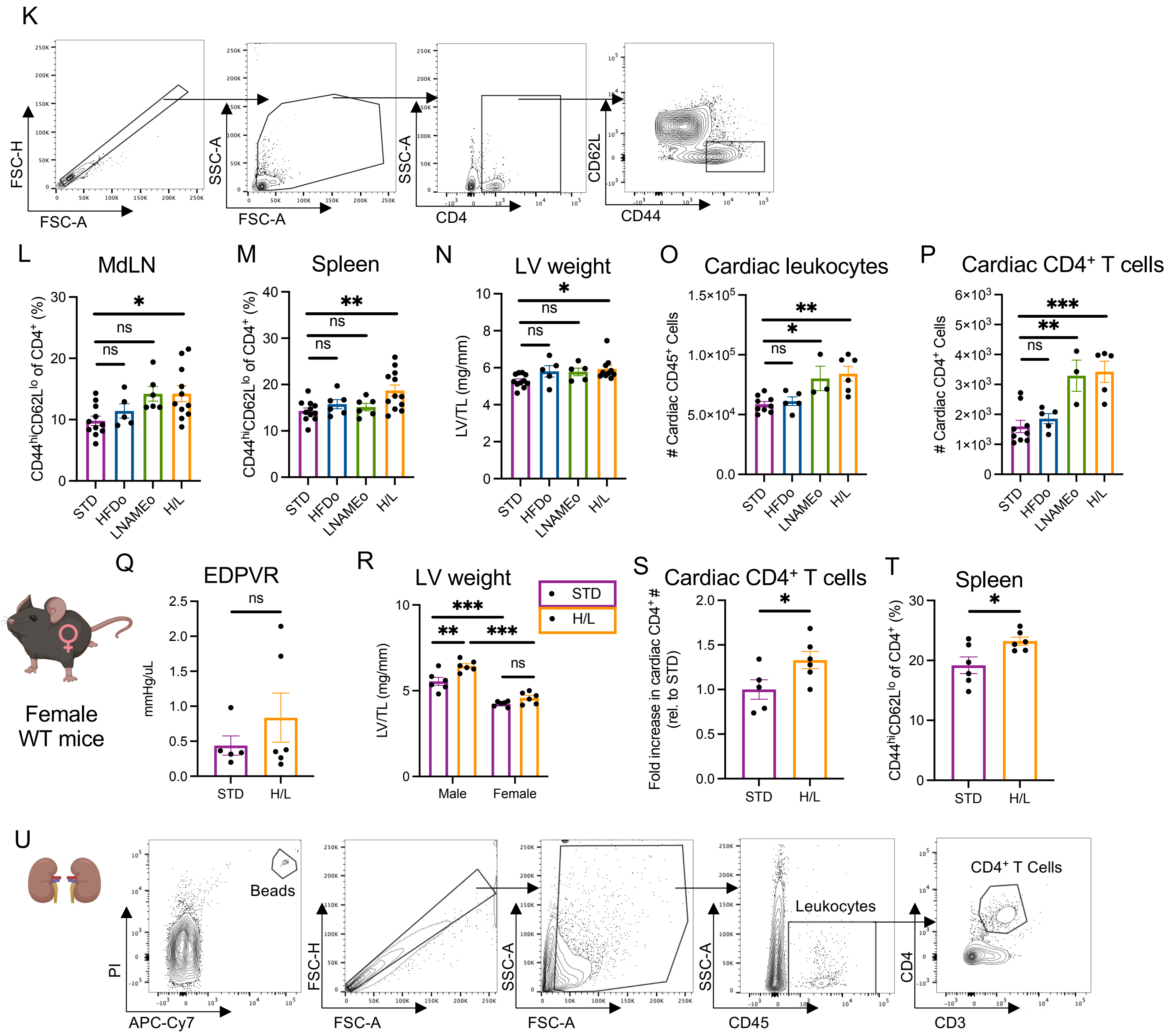

Supplemental Figure 1: Gating strategies for leukocyte characterization by flow cytometry, and characterizing inflammation in female mice and single diet controls. WT mice were fed H/L or STD for 5 weeks. Lymphocytes (A) were measured by flow cytometry, and CD48$^+$ T cell infiltration was visualized in LV (B). Leukocytes (C), CD45$^+$CD11b$^+$ cells (D), CD45$^+$CD11b$^+$Ly6G$^-$ cells (E), and CD45$^+$CD11b$^+$Ly6G$^+$ cells (F) in LV of mice were measured directly by flow cytometry. Cardiac gene expression of *Nlrp3* was measured by qPCR (G). CD45$^+$B220$^+$ cells in LV of mice were measured directly by flow cytometry (H). LV CD45$^+$CD4$^+$ cells expressing IL-4, IFNγ, IL-10, and IL-17 (I-J) were analyzed by flow cytometry. CD4$^+$CD44^{hi}CD62L^{lo} effector T cells were measured in mediastinal lymph nodes and spleen (K) by flow cytometry. WT mice fed HFD only (HFDo), L-NAME only (LNAMEo), H/L, or STD chow for 5 weeks were analyzed for lymphoid expansion of CD4$^+$CD44^{hi}CD62L^{lo} effector T cells (L-M). LV hypertrophy was measured (normalizing LV weight to tibia length, LV/TL, N), and cardiac CD45$^+$ cells (O) and CD45$^+$CD3$^+$CD4$^+$ cells (P) were analyzed directly in LV by flow cytometry. Female mice were fed H/L or STD for 5 weeks and invasive hemodynamic analysis were used to characterize end diastolic pressure volume relationship (EDPVR, Q). Normalized LV weight was used to measure hypertrophy (LV/TL, R), and cardiac CD4$^+$ cells (S) and splenic effector CD4$^+$ cells (T) were measured by flow cytometry. In male WT mice (U), renal CD45$^+$ cells and CD4$^+$ cells were measured by flow cytometry. n=4-12. B-J, W, S-T: unpaired t test. L-P: one-way ANOVA with Tukey's multiple comparisons test. R: two-way ANOVA with Sidak's multiple comparisons test. L-M: HFDo, LNAMEo data are shown compared to STD, H/L data from Figure 1. *$p \leq 0.05$, **$p \leq 0.01$, ***$p \leq 0.001$.

Supplementary Figure 2

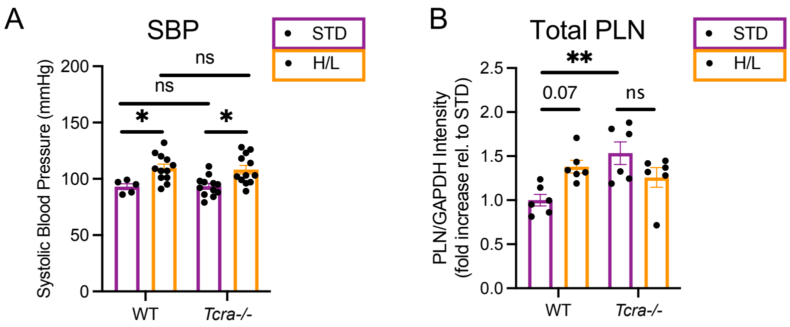

Supplemental Figure 2: Comparison of WT vs *Tcra-/-* fed HFD/L-NAME or STD diet. WT and T cell deficient (*Tcra-/-*) mice were fed STD or H/L for 5 weeks and systolic blood pressure (SBP, A) was measured by invasive hemodynamic analysis. Total phospholamban (PLN) protein expression was assessed in left ventricle (LV) samples by Western blot (B). n=6-12. Two-way ANOVA with Sidak's multiple comparisons test. *p≤0.05, **p≤0.01.

Supplementary Figure 3

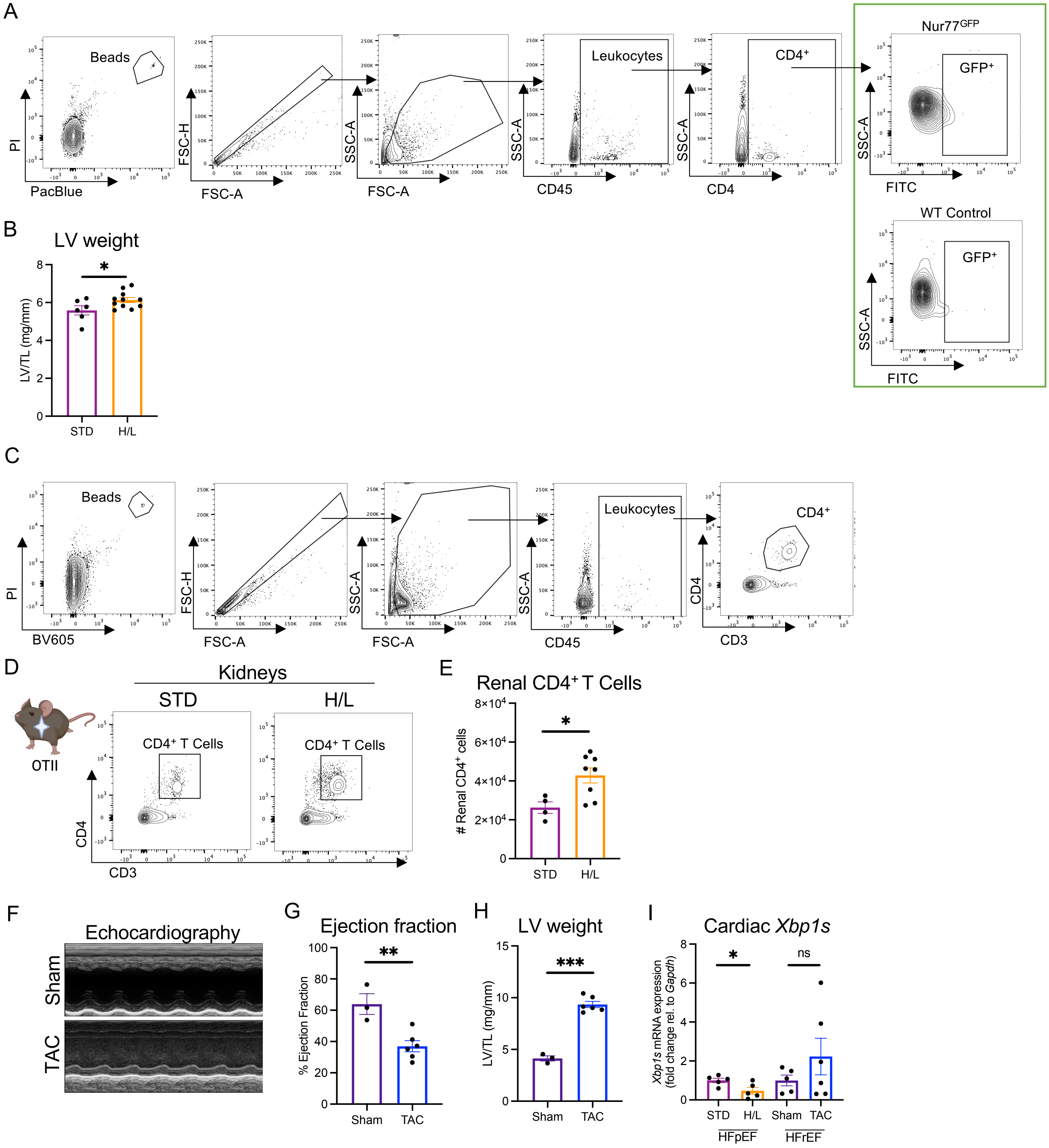

Supplementary Figure 3 continued

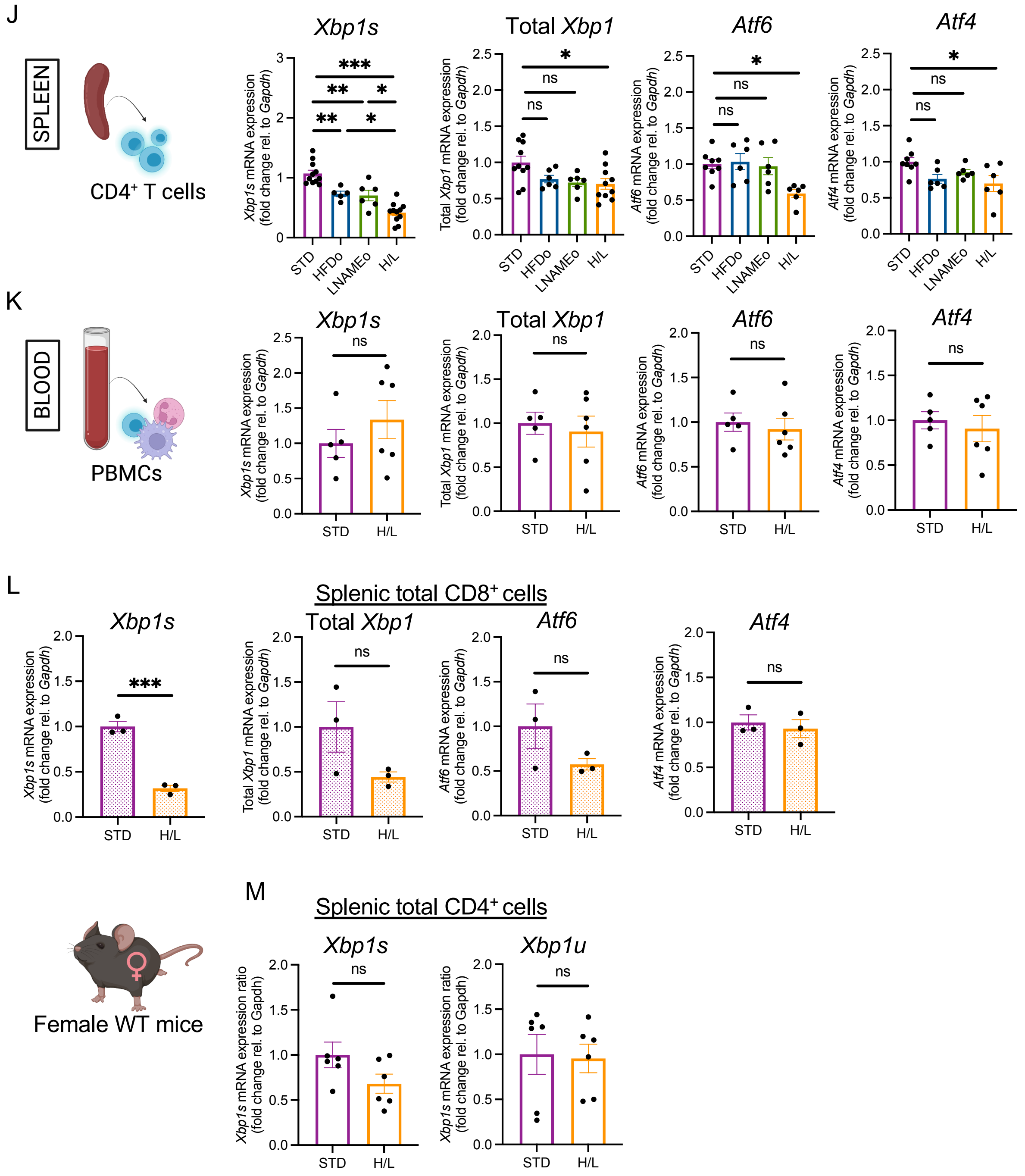

Supplemental Figure 3: Gating strategies for Nur77^{GFP} and OTII mice and leukocyte UPR expression. CD45$^+$CD4$^+$GFP$^+$ cells were measured in LV of Nur77^{GFP} mice 5 weeks after H/L, STD, or TAC surgery by flow cytometry, using a WT mouse as a negative control for gating (A). Left ventricular hypertrophy was measured in Nur77^{GFP} mice by normalizing LV weight to tibial length (LV/TL, B). CD45$^+$CD3$^+$CD4$^+$ cells in LV (C) or in kidneys (D-E) of OTII mice were directly measured by flow cytometry. WT mice underwent TAC or sham surgery, and 5 weeks later, ejection fraction was assessed by echocardiography (F-G) and LV weight was measured (normalized to tibia length, LV/TL, H). Cardiac LV expression of *Xbp1s* was measured by qPCR from mice fed H/L or STD, or receiving TAC or sham surgery, for 5 weeks (I). WT mice were fed HFD only (HFDo), L-NAME only (LNAMEo), H/L, or STD for 5 weeks, then splenic CD4$^+$ T cells were isolated to measure *Xbp1s*, total *Xbp1*, *Atf6*, and *Atf4* expression by qPCR (J). In WT mice fed H/L or STD for 5 weeks, the circulating leukocyte population in blood (K) and splenic CD8$^+$ T cell population (L) were analyzed for expression of *Xbp1s*, total *Xbp1*, *Atf6*, and *Atf4* by qPCR. Splenic CD4$^+$ T cells were isolated from female WT mice fed H/L or STD for 5 weeks, and expression of *Xbp1s* and total *Xbp1* were analyzed by qPCR (M). n=3-11. K-L: each replicate is n=2 mice pooled. B, E-H, K-M: unpaired t test. I: HF groups (H/L or TAC) are compared against their respective controls (STD and sham, respectively) in unpaired t test. J: one way ANOVA with Tukey's multiple comparisons test, and HFDo, LNAMEo groups are shown compared to STD, H/L groups in Figure 3. *$p \leq 0.05$, **$p \leq 0.01$, ***$p \leq 0.001$.

Supplementary Figure 4

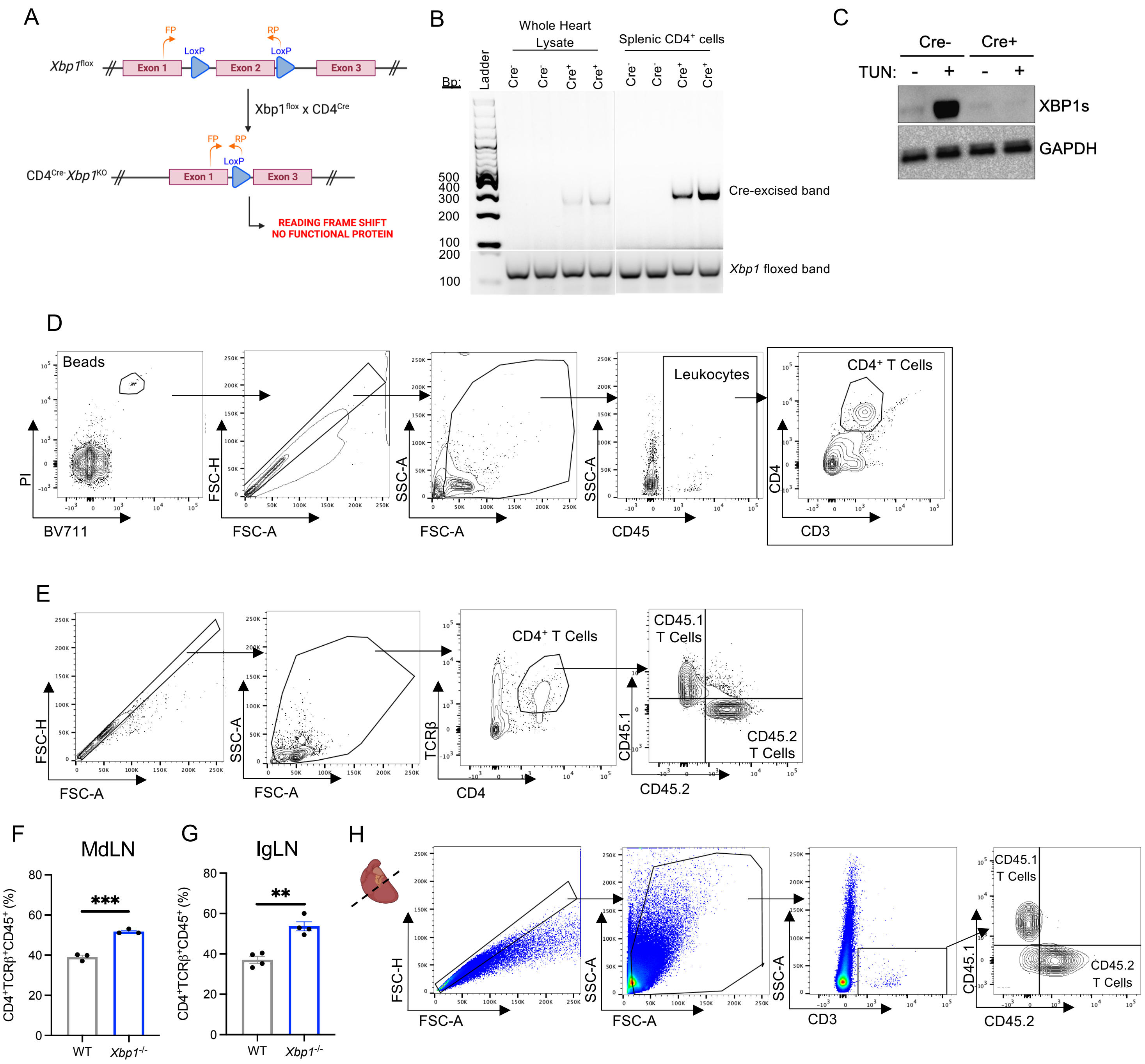

Supplemental Figure 4: Genotyping CD4^{Cre}-*Xbp1*^{flox} mice and gating strategies for cardiac and lymphoid leukocyte populations. CD4^{Cre}-*Xbp1*^{flox} mice were generated by breeding *Xbp1*^{flox} and CD4^{Cre} mice, generating progeny with nonfunctional XBP1 protein (A). Cre excision in splenic CD4^{+} T cells was present in Cre^{+} mice, and absent in Cre^{-} mice (B). Splenic CD4^{+} T cells were isolated from CD4^{Cre}-*Xbp1*^{flox} (T-X*bp1*^{KO}) and CD4^{WT}-*Xbp1*^{flox} (T-X*bp1*^{WT}) mice and expanded into T cell blasts in the presence of αCD3/αCD28. Cells were either left untreated or treated with tunicamycin (TUN) for 6 hours, then collected for analysis of XBP1s expression by Western blotting (C). Cardiac CD45^{+}CD3^{+}CD4^{+} T cells were analyzed in LV of T-X*bp1*^{KO} or T-X*bp1*^{WT} mice directly using flow cytometry (D). CD45.2^{+}CD4^{+}Tcrβ^{+}Xbp1$^{-/-}$ and CD45.1^{+}CD4^{+}Tcrβ^{+}WT T cells were co-transferred into T cell deficient (*Tcra-/-*) recipients fed H/L for 3 weeks, then respective populations of T cells were directly measured in lymphoid organs using flow cytometry (E). Relative proportions of each cell type were characterized in mediastinal lymph nodes (MdLN, F), and inguinal lymph nodes (IgLN, G) of recipient *Tcra-/-* mice by flow cytometry. Relative proportions of CD3^{+}CD45.1^{+} and CD3^{+}CD45.2^{+} cells were measured in left ventricles of *Tcra-/-* mice (H). n=3-4. Unpaired t test. $^{**}p \leq 0.01$, $^{***}p \leq 0.001$

Supplementary Figure 5

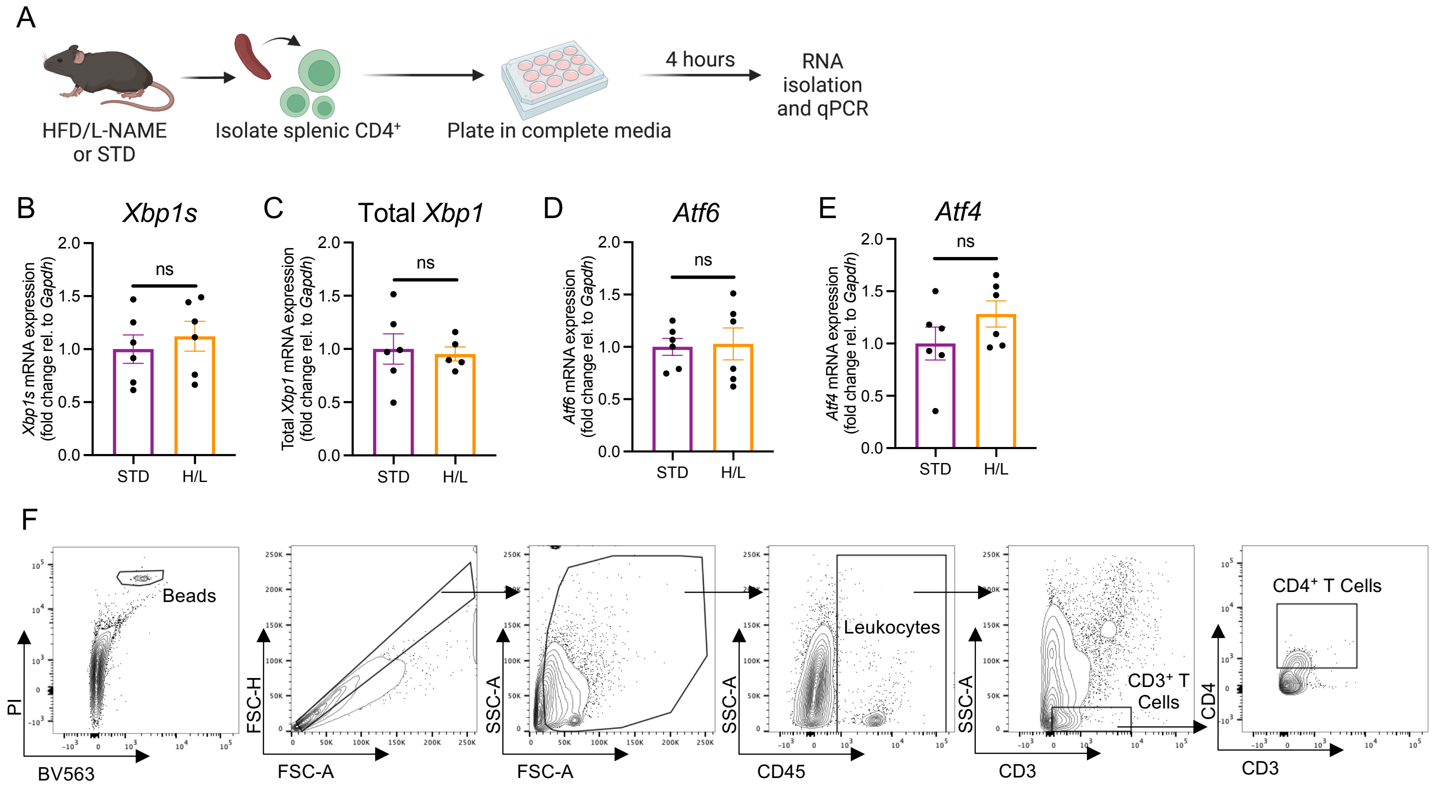

Supplemental Figure 5: UPR recovery in vitro and in vivo. Splenic $CD4^+$ T cells were isolated from WT mice fed H/L or STD for 5 weeks, plated in complete media for 4 hours in 37°C, then measured for gene expression of *Xbp1s,* total *Xbp1*, Atf6 and *Atf4* by qPCR (A-E). Cardiac $CD45^+CD3^+CD4^+$ T cells were directly measured by flow cytometry in WT mice were fed STD or H/L, or H/L for 5 weeks then reverted to STD for 2 weeks (H/L->S) prior to analysis. n=5-6. Unpaired t test.

Supplemental Tables

Supplementary Table 1: Characterization of left ventricular function of female WT mice by echocardiography or pressure volume loops after 5 week treatment with STD or H/L

	Ejection Fraction (%)	Fractional Shortening (%)	Anterior Wall Thickness (mm)	Internal Diastolic Diameter (mm)	Internal Systolic Diameter (mm)	Posterior Wall Thickness (mm)	Heart Rate (bpm)	Systolic Blood Pressure (mm Hg)	dP/dt min (mmHg/s)	dP/dt max (mmHg/s)	EDPVR	ESPVR	PRSW
**WT STD**	59.23 ± 2.048	30.76 ± 1.418	0.793 ± 0.029	3.518 ± 0.046	2.437 ± 0.067	1.174 ± 0.037	478.3 ± 12.2	95.4 ± 1.66	-6142 ± 437.2	6759 ± 333.1	0.439 ± 0.138	2.489 ± 0.357	38.73 ± 6.363
**WT H/L**	62.25 ± 1.216	32.64 ± 0.806	0.925 ± 0.068	3.319 ± 0.074*	2.237 ± 0.068	1.295 ± 0.048	492.3 ± 13.26	120 ± 2.817***	-7048 ± 620.9	7377 ± 730.1	0.837 ± 0.351	2.674 ± 0.511	39.47 ± 8.692

Values are means ± SEM. n=7 for echocardiography; n=7-9 for pressure volume loops. Unpaired t-test. *** ≤ 0.001 comparing STD vs H/L

Supplementary Table 2: Characterization of left ventricular function of WT or *Tcra-/-* mice by echocardiography or pressure volume loops after 5 week treatment with STD or H/L

	Fractional Shortening (%)	Anterior Wall Thickness (mm)	Internal Diastolic Diameter (mm)	Internal Systolic Diameter (mm)	Posterior Wall Thickness (mm)	Heart Rate (bpm)	dP/dt min (mmHg/s)	dP/dt max (mmHg/s)	ESPVR	PRSW
**WT STD**	36.831 ± 3.020	0.822 ± 0.061	3.592 ± 0.13	2.29 ± 0.166	0.768 ± 0.39	473.111 ± 17.501	-8172.2 ± 497.635	8878 ± 551.957	2.508 ± 0.302	59.618 ± 8.555
**WT H/L**	35.266 ± 1.749	0.976 ± 0.047	3.578 ± 0.1	2.331 ± 0.121	0.854 ± 0.028	520.667 ± 10.761*	-8964.9 ± 562.069	8880.9 ± 441.473	5.343 ± 1.699	51.915 ± 5.105
***Tcra-/-* STD**	35.880 ± 1.444	0.852 ± 0.046	3.86 ± 0.117	2.483 ± 0.119	0.806 ± 0.027	496.778 ± 11.351	-5523.2 ± 232.392$$	6190.8 ± 293.030$$	2.713 ± 0.589	47.458 ± 6.893
***Tcra-/-* H/L**	33.534 ± 1.595	0.925 ± 0.04	3.544 ± 0.091	2.364 ± 0.107	0.854 ± 0.022	488.7 ± 8.234	-8458.44 ± 359.889	8622.889 ± 420.918	1.597 ± 0.207	52.726 ± 6.001

Values are means ± SEM. n=9-12 for echocardiography; n=5-10 for pressure volume loops. Two-way ANOVA with Tukey's multiple comparisons test. * ≤ 0.05 comparing WT STD vs H/L; $$ ≤ 0.01 comparing WT vs *Tcra-/-* STD

Supplementary Table 3: Characterization of left ventricular function of OTII mice by echocardiography or pressure volume loops after 5 week treatment with STD or H/L

	Ejection Fraction (%)	Fractional Shortening (%)	Anterior Wall Thickness (mm)	Internal Diastolic Diameter (mm)	Internal Systolic Diameter (mm)	Posterior Wall Thickness (mm)	Heart Rate (bpm)	Systolic Blood Pressure (mm Hg)	dP/dt min (mmHg/s)	dP/dt max (mmHg/s)	EDPVR	ESPVR	PRSW
**OTII STD**	62.60 ± 1.402	33.33 ± 0.978	0.8090 ± 0.038	3.884 ± .07338	2.592 ± 0.2104	0.6859 ± .05153	442.3 ± 42.31	97.14 ± 2.849	-6850 ± 447.2	7322 ± 576.2	0.427 ± 0.164	2.722 ± 0.43	42.13 ± 6.575
**OTII H/L**	66.41 ± 2.037	36.11 ± 1.636	0.9237 ± 0.1378	3.665 ± 0.0668*	2.345 ± 0.2338	0.8344 ± 0.03616*	495.6 ± 51.68*	113.9 ± 2.201***	-8094 ± 303.6*	7895 ± 334.8	0.371 ± 0.132	2.501 ± 0.4371	50.57 ± 5.849

Values are means ± SEM. n=7 for echocardiography; n=7-9 for pressure volume loops. Unpaired t-test. * ≤ 0.05, *** ≤ 0.001 comparing STD vs H/L

Supplementary Table 4: Characterization of left ventricular function of WT mice by echocardiography 5 weeks after TAC or sham surgery

	Fractional Shortening (%)	Anterior Wall Thickness (mm)	Internal Diastolic Diameter (mm)	Internal Systolic Diameter (mm)	Posterior Wall Thickness (mm)	Heart Rate (bpm)
**WT Sham**	34.28 ± 5.016	0.8529 ± 0.088	3.252 ± 0.1696	2.141 ± 0.3881	0.7008 ± 0.0323	488.3 ± 59.5
**WT TAC**	17.80 ± 1.941**	1.086 ± 0.058*	4.409 ± 0.0936***	3.63 ± 0.3586***	0.9833 ± 0.0548*	520.8 ± 49.49

Values are means ± SEM. n=3-6 for echocardiography. Unpaired t-test. $^{*} \leq 0.05$, $^{**} \leq 0.01$, $^{***} \leq 0.001$ comparing sham vs TAC.

Supplementary Table 5: Characterization of left ventricular function of T-*Xbp1*^{WT} and T-*Xbp1*^{KO} mice by echocardiography or pressure volume loops after 5 week treatment with STD or H/L

	**Fractional Shortening (%)**	**Anterior Wall Thickness (mm)**	**Internal Diastolic Diameter (mm)**	**Internal Systolic Diameter (mm)**	**Posterior Wall Thickness (mm)**	**Heart Rate (bpm)**	**dP/dt min (mmHg/s)**	**dP/dt max (mmHg/s)**	**ESPVR**	**PRSW**
**T-*Xbp1*^{WT} H/L**	29.63 ± 1.217	0.946 ± 0.0534	3.76 ± 0.1118	2.645 ± 0.1536	0.9080 ± 0.061	526.3 ± 34.11	-8412 ± 812.0	8554 ± 492.3	1.126 ± 0.524	42.65 ± 5.707
**T-*Xbp1*^{KO} H/L**	34.55 ± 1.931	1.060 ± 0.1067	3.557 ± 0.1050	2.330 ± 0.2337	1.059 ± 0.0579	508.8 ± 37.85	-7414 ± 276.1	7307 ± 172.2*	0.7312 ± 0.2380	48.18 ± 9.814

Values are means ± SEM. n=4 for echocardiography; n=4-6 for pressure volume loops. Unpaired t-test. * ≤ 0.05 comparing *Xbp1*^{WT} vs *Xbp1*^{KO}

Supplementary Table 6: Characterization of left ventricular function of WT mice by echocardiography or pressure volume loops after treatment with STD, H/L, or H/L then STD for 2 weeks (2 STD) or 3 weeks (3 STD).

	Ejection Fraction (%)	Fractional Shortening (%)	Anterior Wall Thickness (mm)	Internal Diastolic Diameter (mm)	Internal Systolic Diameter (mm)	Posterior Wall Thickness (mm)	Heart Rate (bpm)	dP/dt min (mmHg/s)	dP/dt max (mmHg/s)	ESPVR	PRSW
**WT STD**	63.71 ± 1.675	34.10 ± 1.160	0.9438 ± 0.043	3.822 ± 0.1462	2.525 ± 0.3251	0.7998 ± 0.007	498.5 ± 16.23	-7743 ± 596.5	7671 ± 450.7	1.820 ± 0.2144	34.52 ± 6.813
**WT H/L**	63.45 ± 2.883	33.80 ± 2.037	0.9304 ± 0.077	3.472 ± 0.142	2.306 ± 0.3539	0.8369 ± 0.0203	482.5 ± 10.23	-7406 ± 249.8	8122 ± 116.3	9.353 ± 3.344	43.28 ± 5.316
**WT H/L -> 2 STD**	59.74 ± 2.861	32.07 ± 2.443	0.8820 ± 0.066	3.728 ± 0.092	2.556 ± 0.214	0.8142 ± 0.0238	474.5 ± 20.02	-5403 ± 340.3$^{\$}$	5928 ± 345.5$^{\$}$	5.662 ± 2.917	33.96 ± 11.83
**WT H/L -> 3 STD**	60.04 ± 2.130	31.55 ± 1.397	0.898 ± 0.057	3.877 ± 0.105	2.659 ± 0.116	0.924 ± 0.061	524.2 ± 8.94$^{\%\%,\wedge\wedge\wedge}$	-7805 ± 686.7$^{\wedge}$	8867 ± 687.5$^{\wedge\wedge}$	1.961 ± 0.5288	43.7 ± 9.448

Values are means ± SEM. n=6 for echocardiography; n=5-6 for pressure volume loops. One-way ANOVA with Tukey's multiple comparisons test. $^{\$}$ ≤ 0.05 comparing H/L vs H/L->2STD; $^{\%\%}$ ≤ 0.01 comparing H/L vs H/L->3STD; $^{\wedge}$ ≤ 0.05, $^{\wedge\wedge}$ ≤ 0.01, $^{\wedge\wedge\wedge}$ ≤ 0.001 comparing H/L->2STD vs H/L->3STD.

Supplementary Table 7: Antibodies used for flow cytometry

Target	Clone	Biolegend Catalog #
CD45.2	104	109841
CD3	145-2C11	100330
CD4	GK1.5	100412
CD8	53-6.7	100738
CD11b	M1/70	101230
Ly6G	1A8	127628
IL-4	11B11	504109
IFNγ	XMG1.2	505809
IL-17	TC11-18H10.1	506904
IL-10	JES5-16E3	505022
CD44	IM7	103012
CD62L	MEL-14	161204
CD45.1	A20	110708
TCRβ	H57-597	109220
CD69	H1.2F3	104506

Supplementary Table 8: Primer sequences used for qPCR

Gene	Forward (5' – 3')	Reverse (5' – 3')
Xbp1s	GGTCTGCTGAGTCCGCAGCAGG	GAAAGGGAGGCTGGTAAGGAAC
Total *Xbp1*	GACAGAGAGTCAAACTAACGTGG	GTCCAGCAGGCAAGAAGGT
Atf6	AGAAAGCCCGCATTCTCCAG	ACTCCCAGAATTCCTACTGATGC
Atf4	ATGGCCGGCTATGGATGAT	CGAAGTCAAACTCTTTCAGATCCATT
Nlrp3	ATTACCCGCCCGAGAAAGG	CATGAGTGTGGCTAGATCCAAG
Gapdh	TGGCAAAGTGGAGATTGTTGCC	AAGATGGTGATGGGCTTCCCG
Xbp1 (splicing assay)	ACACGCTTGGGAATGGACAC	CCATGGGAAGATGTTCTGGG
18s (splicing assay)	AAACGGCTACCACATCCAAG	CCTCCAATGGATCCTCGTTA

Supplementary Table 9: Antibodies used for Western blot

Target	Catalog #
Phospho-IRE1α (Ser724)	Novus NB100-2323
IRE1α	Cell Signaling 3294T
Phospho-PERK (Thr980)	Cell Signaling 3179S
PERK	Cell Signaling 3192S
Phospho-EIF2α	Cell Signaling 9721
EIF2α	Cell Signaling 9722
ATF6	Cell Signaling 65880
ATP2A2/SERCA2	Cell Signaling 4388
Phospho-Phospholamban (Ser16/Thr17)	Cell Signaling 8496
Phospholamban	Cell Signaling 14562
GAPDH	Cell Signaling 2118S
